# JAK‐STAT core cancer pathway: An integrative cancer interactome analysis

**DOI:** 10.1111/jcmm.17228

**Published:** 2022-03-01

**Authors:** Fettah Erdogan, Tudor Bogdan Radu, Anna Orlova, Abdul Khawazak Qadree, Elvin Dominic de Araujo, Johan Israelian, Peter Valent, Satu M. Mustjoki, Marco Herling, Richard Moriggl, Patrick Thomas Gunning

**Affiliations:** ^1^ 71637 Department of Chemical and Physical Sciences University of Toronto Mississauga Mississauga Ontario Canada; ^2^ Department of Chemistry University of Toronto Toronto Ontario Canada; ^3^ Institute of Animal Breeding and Genetics University of Veterinary Medicine Vienna Austria; ^4^ 27271 Division of Hematology and Hemostaseology Department of Internal Medicine I Medical University of Vienna Vienna Austria; ^5^ 27271 Ludwig Boltzmann Institute for Hematology and Oncology Medical University of Vienna Vienna Austria; ^6^ 3835 Translational Immunology Research Program and Department of Clinical Chemistry and Hematology University of Helsinki Helsinki Finland; ^7^ Hematology Research Unit Helsinki University Hospital Comprehensive Cancer Center Helsinki Finland; ^8^ iCAN Digital Precision Cancer Medicine Flagship Helsinki Finland; ^9^ Department of Hematology, Cellular Therapy, and Hemostaseology University of Leipzig Leipzig Germany

**Keywords:** blood cancer, breast cancer, colorectal cancers, JAK/STAT pathway in cancers, liver cancers, lung cancer, protein‐protein interactions, systems medicine

## Abstract

Through a comprehensive review and in silico analysis of reported data on STAT‐linked diseases, we analysed the communication pathways and interactome of the seven STATs in major cancer categories and proposed rational targeting approaches for therapeutic intervention to disrupt critical pathways and addictions to hyperactive JAK/STAT in neoplastic states. Although all STATs follow a similar molecular activation pathway, STAT1, STAT2, STAT4 and STAT6 exert specific biological profiles associated with a more restricted pattern of activation by cytokines. STAT3 and STAT5A as well as STAT5B have pleiotropic roles in the body and can act as critical oncogenes that promote many processes involved in cancer development. STAT1, STAT3 and STAT5 also possess tumour suppressive action in certain mutational and cancer type context. Here, we demonstrated member‐specific STAT activity in major cancer types. Through systems biology approaches, we found surprising roles for EGFR family members, sex steroid hormone receptor ESR1 interplay with oncogenic STAT function and proposed new drug targeting approaches of oncogenic STAT pathway addiction.

## 1 INTRODUCTION

**FIGURE 1:**
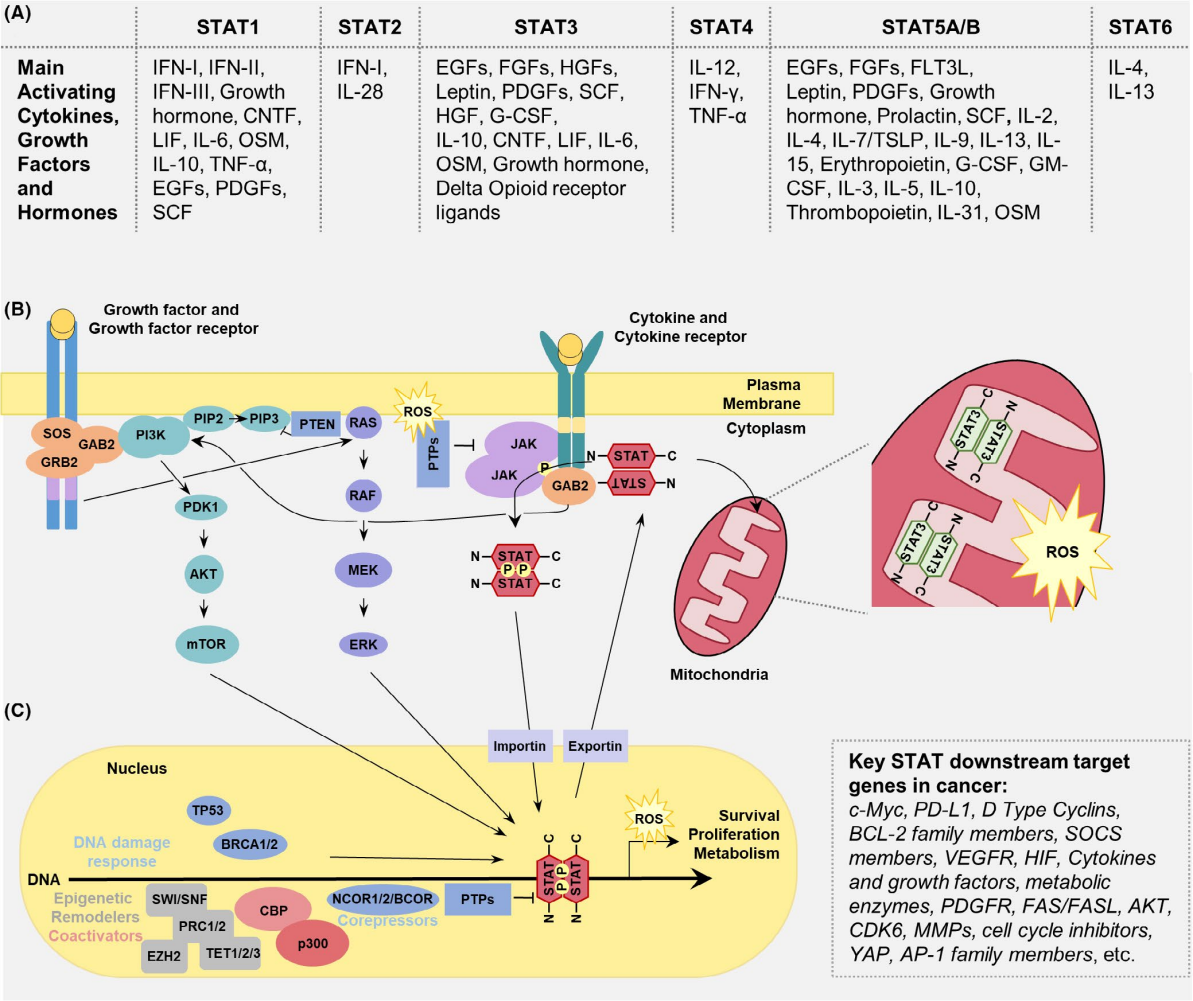
Common STAT cellular activation‐inactivation cycle pathways. (A) Variety of cytokines, hormones and growth factors can activate STAT family members. (B) Binding of the ligand to either the cytokine or growth factor (GF) receptor induces series of the activation cascades. Whereas JAK kinase activation is generally exclusive to cytokine receptor signaling, STAT action is triggered by both receptor types. Typically, auto‐phosphorylated receptor‐associated Janus kinases (JAKs) phospho‐activate STATs which form STAT parallel dimer and are transported across nuclear membrane by Importin α’s (KPNA1) and involve the action of Nucleophosmin I decamer (not shown). Binding of activated STATs to DNA is coupled to interactions with various transcriptional regulators discussed in the main text. Phosphatase‐mediated inactivation of the DNA‐bound STAT complex disengages the dimer from the DNA, breaks them into monomers and leads to their export via nuclear exportins (XPO1). Cytokine and growth factor receptors are both inducers of the RAS‐RAF‐MAPK and the PI3K‐AKT‐mTOR pathways. For simplicity, other signaling pathways such as activation of PKC or PLC are excluded. STAT3 is also capable of localizing into the mitochondria inner matrix and influencing ROS production. (C) STATs are also reported to be involved in the DNA damage response (e.g., TP53 and BRCA1/2) and interact with epigenetic modifiers (e.g., EZH2, TET1/2/3, SWI/SNF, PRC1/2, CBP‐p300 and NCOA‐1). CNTF, ciliary neurotrophic factor; EGF, Epidermal growth factor; FGF, fibroblast growth factor; FLT3L, FMS‐like tyrosine kinase 3 ligand; G‐CSF, granulocyte colony‐stimulating factor; GM‐CSF, granulocyte‐macrophage colony‐stimulating factor; HGF, hepatocyte growth factor; IFN, interferon; IL, interleukin; LIF, leukemia inhibitory factor; OSM, oncostatin M; PDGF, platelet‐derived growth factor; SCF, stem cell factor; TGF, transforming growth factor; TNF, tumor necrosis factor; TSLP, thymic stromal lymphopoietin

Transcription factors are the gatekeepers of cellular processes. They control the expression of genes encoding critical proteins of the entire proteome, from proteins involved in metabolism and cell communication to those that regulate the immune response and cell cycle. Signal transducer and activator of transcription (STAT) refers to a family of seven transcription factors that regulate the expression of genes controlling critical cellular processes in a mechanism distinct from secondary messengers.^1^, ^2^ Activation of STATs is typically initiated by ligand binding at cell surface receptors, followed by kinase‐dependent phosphorylation of a conserved tyrosine (pY) residue.^3^ This leads to Src 2 homology (SH2) domain mediated STAT‐STAT parallel dimerization, rapid translocation into the nucleus, DNA binding and subsequent transcription of gene targets^4^, ^5^, ^6^ (Figure 1).

All STAT members (Figure 1) display similar biochemical features controlling their subcellular localization and mode of action. Recent studies revealed that not only do they transduce signals to control transcription, they also regulate anabolism and catabolism at the mitochondria and are involved in nuclear compartmentalisation and genome integrity. Cytokine or growth factor signalling and their activation, however, is largely cell type dependent. Although cytokine or growth factor interactions with cellular components such as STATs are broadly deemed as protein–protein interactions, their effective mechanism is incompletely understood. Here, we investigate the STAT interactome in a gene product and cancer type‐specific manner. Depending on the biological processes involved, the presence of cytokines, and underlying conditions and pathologies, they have a unique STAT interactome that illuminates the pleiotropic action of STAT family members. Numerous reports have shown that gain‐of‐function (GOF) mutations (i.e., somatic or acquired variations) in STAT proteins are a basis for oncogenesis in neoplastic cells.^7^, ^8^, ^9^, ^10^, ^11^

Our investigation focussed on the five major cancers ranked based on death rate, namely lung, breast, prostate, colorectal and liver cancers. Due to a high number of driver mutations in the STAT pathway, we further included blood cancers in our interactome analysis. We initially compiled all literature‐based STAT interactions reported, analysed their member‐specific role and generated a pathway‐based figure depicting common STAT activation pathways. We then filtered STAT protein–protein interactions (PPIs) into those exclusively reported in association with a specific cancer type, either lung, breast, prostate, colorectal or liver cancer^12^ and compared them with the global STAT interactome. We conducted a thorough analysis of the STAT interactome in blood cancers following these solid cancers. Overall, our analyses confirmed the central targets across multiple cancers such as the growth factor receptors (e.g., EGFR), while also revealing new viable targets such as the nuclear hormone receptor (e.g., ESR1) pathways in lung cancers, and supported the application of new single and combinatorial targeted approaches for the treatment of specific cancers.

## 2 MATERIALS AND METHODS

The level of activity and protein interactions of each STAT member varies significantly. In the context of disease and specific cell type, some STAT members have a wider influence as demonstrated by a larger number of PPIs. In order to capture and visualize this influence and their differential activities, we summarized STAT interactions in a network map and segregated their PPIs into disease and tissue contexts. PPI data for most human proteins can be effectively used to identify pathogenic genes, drug targets and drug efficacy.^13^, ^14^, ^15^, ^16^, ^17^, ^18^ These data were collected using the integrated interactions database (IID) 2018 version (http://iid.ophid.utoronto.ca/)^19^. The IID gathers PPIs from nine curated databases and segregates these interactions into disease and tissue contexts. IID assigns context based on gene expression, if the mas5 normalized expression >200, with gene expression levels derived from 20 NCBI GEO gene expression datasets.^19^, ^20^, ^21^, ^22^ Given that PPIs are usually monitored in cells or in vitro, two proteins are considered to interact in a specific context if they have been shown to interact in two independent publications/databases/assays, and if both of those genes meet the above gene expression requirements in the specific context (e.g., breast cancer). The interactions presented here are curated to ensure minimum inclusion of false positive and negatives. To minimise the false positive rate, we only report interactions which are present in at least two publications or confirmed by at least two bioassays. To minimise the false negative rate, IID uses high confidence predictions to look for additional interactions.^23^, ^24^ To generate the interactome figures used here, we input the proteins we were interested in (STATs) into the Enter IDs field and retrieved all PPIs of the STATs in humans. We included experimental, predicted and protein orthology interactions, with thresholds for 2 or more studies, or two or more bioassays. We exported these data with source info, disease and subcellular localization context labelled. The data were then imported into NAViGaTOR 3, a network analysis software developed for protein–protein interactions (http://navigator.ophid.utoronto.ca/navigatorwp/ ^25^ which was used to generate the interactome maps.

**FIGURE 2:**
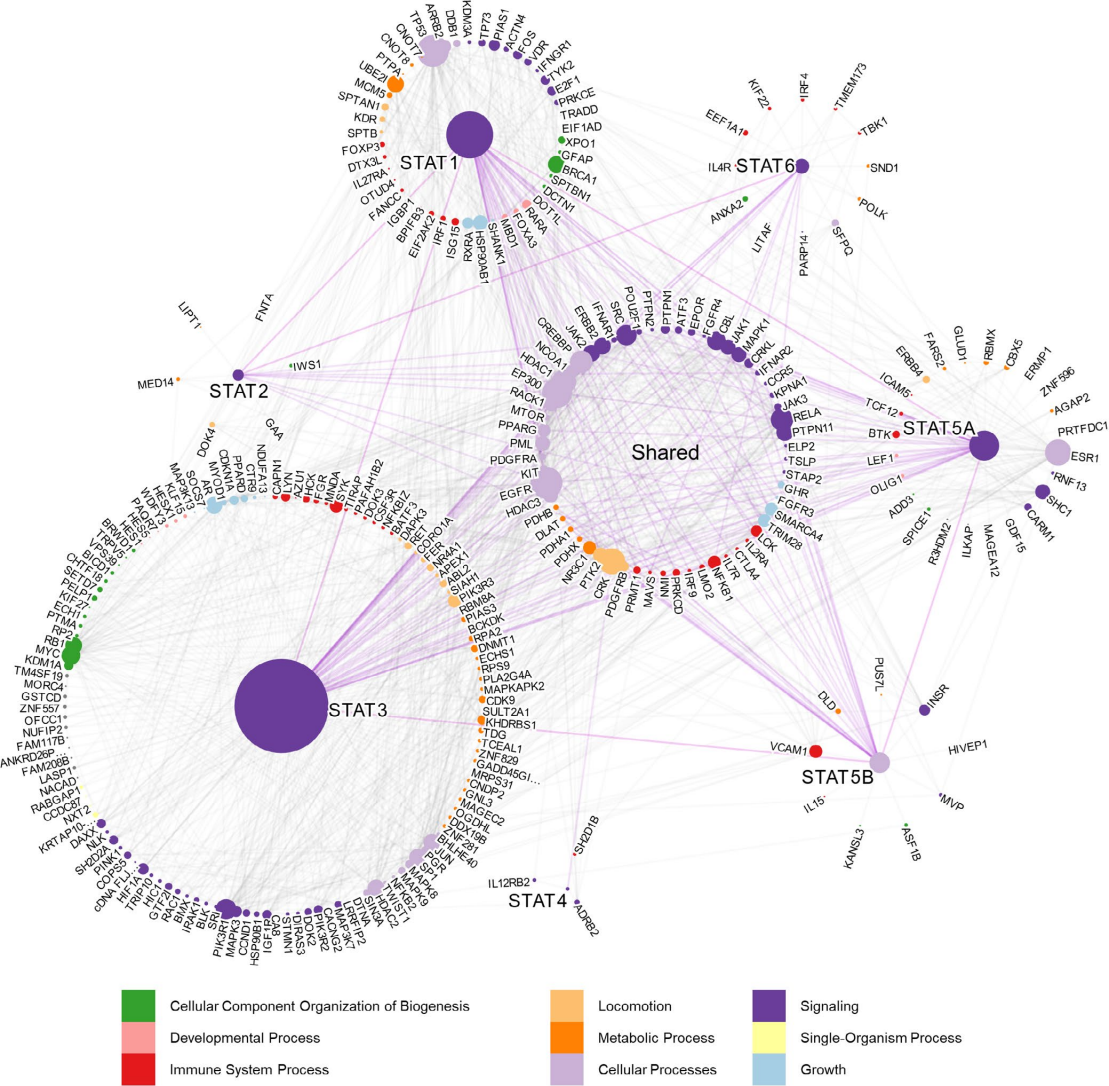
STAT interactome. Global STAT interactome presenting all literature‐based STAT PPIs. Each node (dot) is a protein, and each edge (line) is an interaction between two proteins. The size of the nodes is proportional to their degree, and the color of each node is representative of the biological process of the protein as described in the legend. The length of the edges connecting each node is arbitrary. Proteins which were found to interact with multiple STATs are grouped in the middle, and those exclusive to one STAT are grouped around that corresponding STAT member. A summary table of all interactions can be found in Table S1

**FIGURE 3:**
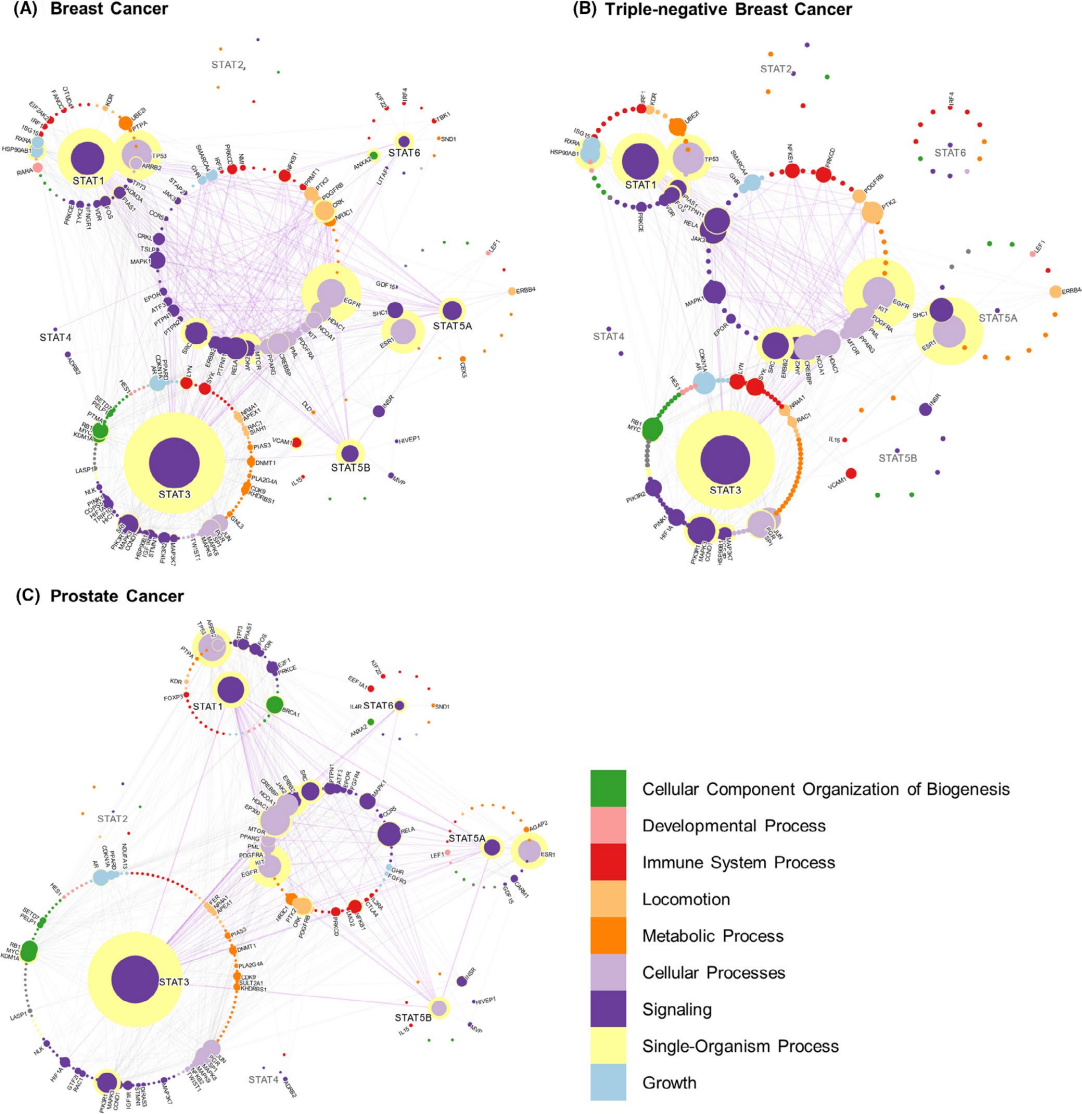
STAT interactions in breast cancer, TNBC and prostate cancer. STAT interactions in two of the five most common death‐causing cancers listed by the WHO; breast cancer and prostate cancer, as well as the breast cancer subtype TNBC. Any interaction shared between two or more STATs is grouped in the middle, and the edges connecting those interactions are highlighted. Proteins for which a label is shown are those which are reported to be over‐expressed in that disease. Although not all proteins directly interact with each other, they may communicate indirectly through hub proteins. The proteins important for indirect communication are identified as ‘central’ to the networks and have a yellow halo in the network maps. Centrality measures how important the protein is for communication between the STAT interactome. It is measured using an undirected all‐pairs shortest path algorithm which measures how many of the shortest paths between proteins pass through a node. Therefore, proteins which are more central act as hubs through which signals are efficiently transmitted from one end of the interactome to the other. The centrality of a protein is proportional to the size of the yellow halo

**FIGURE 4:**
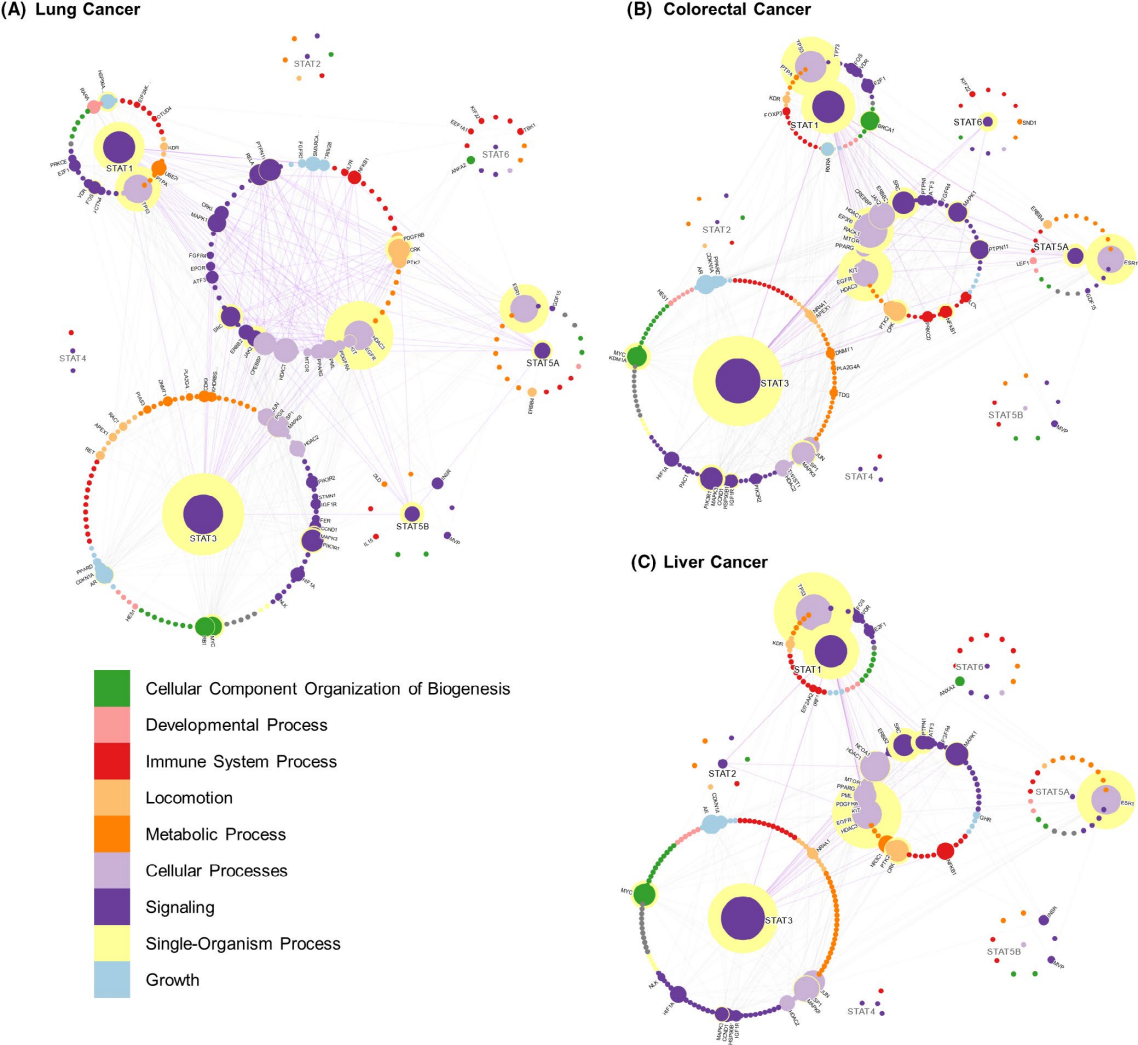
STAT interactions in lung, colorectal and liver cancers. STAT interactions in the three of the five commonly death‐causing cancers listed by the WHO lung cancer, colorectal cancer and liver cancer. For a descriptor of the interactome labels, see Figure 3

**FIGURE 5:**
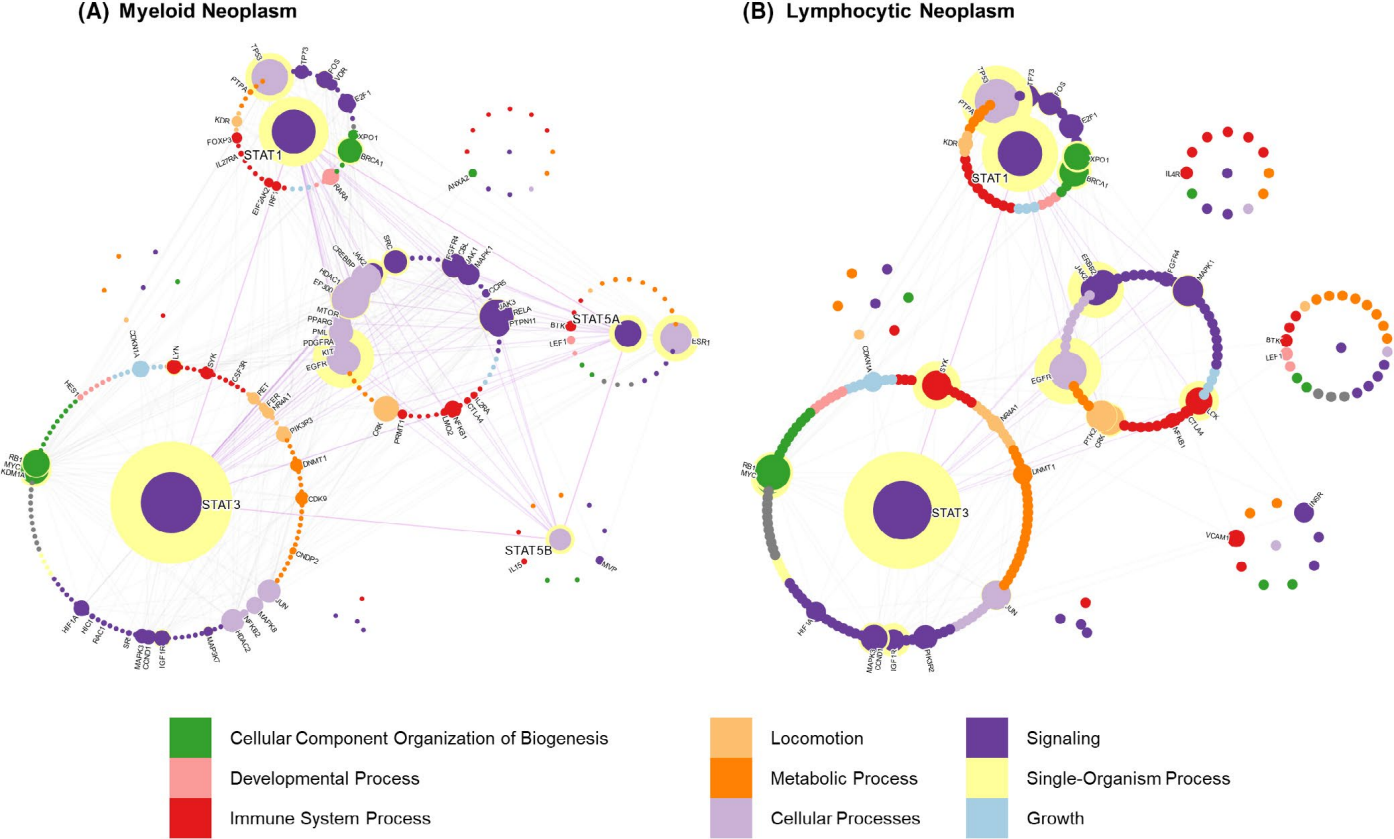
STAT interactions in hematopoietic cancers. STAT interactions in the categories of myeloid and lymphoid neoplasms. For a descriptor of the interactome labels, see Figure 4

Finally, it should be noted that while we can interpret these PPIs to achieve a deeper understanding of STAT communication, care should be taken when interpreting interactions that are not observed. For example, an interaction in the global interactome (Figure 2) can be absent in the context of a certain disease (Figure 3, Figure 4, and Figure 5) due to the rarity of the disease, lack of study analysis interest, lack of sufficient literature support or due to the complexity of given cancer subtypes as illustrated with our breast cancer insights.

To analyse these PPI networks, we examine several factors including the ‘degree’ and ‘centrality’ of each protein with its interaction partners. ‘Degree’ correlates with how many interactions a protein makes, and ‘degree’ also reports on how influential a protein is in its immediate environment. The ‘centrality’—measured through an undirected all pairs shortest paths (APSP) analysis—is indicative of how important a protein is for communication throughout the network. The higher the number of ‘shortest paths pass’ that pass through a protein, the more central it is, and thus, the more important it is for communication between proteins within the network. Together these parameters offer a strong indication of how important a node is to the network, and this can be extrapolated to determine how influential a protein is in signalling pathways, as well as identifying promising therapeutic targets.^13^, ^14^, ^16^, ^17^ IID has been used recently as a part of CoVex to integrate protein–protein interactions into a system which helps identify already approved drugs to treat COVID‐19.^26^, ^27^ A summary table of all interactions, interactor UniProt codes and the Pubmed ID(s) referencing each interaction can be found in Table S1.

## 3 RESULTS AND DISCUSSION

### 3.1 Global STAT interactions

Although all STATs share a role in cell signalling and biogenesis, member‐specific protein interactions reveal divergent activity of STAT members in various biological processes (Figure 2, Figure S1). Compilation of literature‐based data on STAT PPIs shows that STAT3 interacts with the largest number/diversity of proteins (166 distinct proteins), followed closely by STAT1 (81 distinct proteins, Figure 2). The number of publications on a given STAT directly influences its appearance in the interactomes. For example, while STAT1 as a search term results in 10,272 publications, STAT3 results in ~28,419 hits in current PubMed database entry as of 14 July 2021. The search term STAT5 (for STAT5A/B) results only in 6616 hits. STAT3 possesses exclusive and extensive interaction evidence towards oncogenic partners such as c‐Myc, Histone deacetylase 2 (HDAC2) and Mitogen‐activated protein kinase 3 (MAPK3). Other key STAT3‐exclusive binding partners include the Androgen receptor (AR), Spleen tyrosine kinase (SYK) and the P110α/β/γ/δ/P85 regulatory subunits of Phosphatidylinositol 3‐kinase (PI3K). The constant P85 subunit of PI3K is bound by GAB1/2 scaffold proteins that also bridge STAT5A/5B interaction in the cytoplasm, and STAT5 can transcribe an AKT isoform. These interactions, however, were filtered out by our interactome stringency rules. Mutations that lead to increased kinase activity of *PIK3CA* were found in up to 13% of solid cancers including breast, colorectal, lung carcinomas, head and neck cancers among many others. As such, the involvement of STAT1/3/5 family members in the PI3K‐AKT‐mTOR‐S6 pathway is key in these cancers.^28^ All of these proteins also interact with other non‐STAT proteins within the STAT interactome suggesting that while they are directly interacting with STAT3, they are indirectly communicating with other STATs. The fact that some of these interaction partners such as MAPK3 and c‐Myc are important oncogenes indicates that STAT3 could be the vulnerable connector between these oncogenes, which also validates STAT3 as a promising cancer cell target.

Although STAT3 has the largest impact in the interactome, every other STAT also has exclusive interacting partners. STAT1 is largely an antagonist of oncogenic STAT3, also reflected through its function as a heterodimerization partner of STAT3. The balancing role of STAT1 as a tumour suppressor protein over oncogenic STAT3 is well studied in various cancer types, including colorectal cancer.^29^ STAT1 is the most prominent STAT protein interacting with the tumour suppressor TP53 by network analysis, although STAT3 and STAT5A/B have also been reported to interact with TP53 as well.^30^, ^31^

Exclusive interaction of STAT5A and STAT6 with Bruton's tyrosine kinase (BTK) and Interleukin‐4 receptor (IL‐4R) proteins, respectively, highlights another point of member‐specific STAT activity.^32^ The IL‐4Rα chain is also a direct target gene of STAT5,^33^ and both IL‐4 and IL‐13 can activate STAT5 and STAT6.^34^ Surprisingly, STAT5A is the only STAT that has been shown to interact with the Estrogen receptor (ESR1) (Figure 2), and this is thought to occur via the N‐terminal domain of STAT5 in a fashion similar to many other nuclear hormone receptors that drive sex‐specific cancers.^35^, ^36^ ESR1 has a large number of interactions in the global STAT interactome (Figure 2), and it has an important role in female reproductive tract cancers.^37^ These representative key interactions predominantly differentiate the roles of STAT members and increase the diversity of STAT influence on biological processes.

Based on the shared proteins in the core of the interactome (58 proteins), the largest biological processes under which STATs converge are signal transduction or reprogramming of the nucleus, largely through the kinase interactome. Within the shared interactions, most of these proteins are tyrosine kinases such as JAK1/2/3, SRC, MAPK1, EGFR (human Epidermal growth factor receptor; HER1) (Figure S2), and phosphatases such as PTPN11, which is also part of the MAPK pathway, and is involved in signal transduction from the cytoplasm to the nucleus. These signalling proteins are important, as phosphorylation of STATs is a major contributor to their activation, subsequent dimerization, nuclear shuttling or transcriptional elongation. The second largest family of proteins in the core of the interactome are controllers of cellular processes, including coactivators or corepressors of gene expression, cell cycle regulators such as cell cycle inhibitors, cell cycle kinases or cyclins and physiological responses to circadian rhythm that is also controlled largely by GR stress hormone action. Of all the shared interacting partners mentioned above, EGFR and JAK2 are the most central between STAT proteins (Figure S1) and both modulate the STAT signalling cascade through recruitment and phosphorylation of STATs. This is consistent with their reliance on phosphorylation for signalling. Another aspect of cellular processes in which STATs are involved appears to be histone acetylation‐deacetylation. Histone acetylation is an epigenetic marker, which determines DNA transcription kinetics. CBP (CREB‐binding protein), p300 and NCoA‐1 (Nuclear receptor coactivator 1) display histone acetyl transferase activity while HDAC1 and HDAC3 deacetylate histones as well as some non‐histone substrates. Contrastingly, PML (promyelocytic leukaemia) protein is a positive regulator of histone deacetylation and has been shown to interact with STATs in AML. This evidence of multiple positive and negative effectors of histone acetylation suggests that STATs have a large and unobserved role to play in epigenetic regulation.

The shared interactions between STATs further diversify based on their sequence and structure similarities. For example, STAT5A paired with STAT5B, and STAT1 paired with STAT3 have more PPIs in common than any other pair of STATs (Figure S1) and this is supported by their similarities in both the sequence alignment and structural alignment data.^38^ Some of the shared key interaction partners of STAT3 and STAT1 include the mammalian Target of rapamycin (mTOR), HDAC3 and SRC kinase which phosphorylates both STAT1 and STAT3.^39^, ^40^ Importantly, STAT5A and STAT5B are direct phosphorylation targets of mTOR at a conserved S193 residue. Interestingly, STAT1 and STAT3 have more PPIs in common than the more structural homologous STAT5A and STAT5B though, this may again be biased by more literature content available for STAT1/3 than STAT5A/B. It should be noted that partial correlation of STATs having common PPIs does not mean that the two proteins are redundant in function. For example, despite over 90% structural identity, STAT5A has 24 exclusive interactions absent in STAT5B and, STAT5B has 9 exclusive interacting partners with insufficient evidence for STAT5A binding (Figure S1). This indicates that minor changes in a STAT protein could modulate its binding interface and interaction partners.

### 3.2 Disease and tissue‐specific STAT interactions

The plasticity of the five major cancer killers discussed here is mainly due to the biochemical and somatic heterogeneity that manifests in numerous cancer stem cell‐derived subclones during cancer evolution and the tendency of these neoplasms to transdifferentiate and to escape the immune system in various subclones or metastases.^41^ The tumour microenvironment and the status of the immune system play a decisive role in the success rates in cancer therapy.^42^ Single cell sequencing and cancer pathway studies in primary cancer lesions, although conducted with the intention to define the core cancer pathway and simplify cancer for rational targeting efforts, have revealed a highly convoluted picture that awaits further simplification to fully comprehend cancer origins and progression.^43^, ^44^ New drivers, oncogenes, tumour suppressors and transcriptional regulatory elements are uncovered with the aim of adding to our understanding of cancer that has best to be illustrated in a simplified manner. Herein, our concept of disease filtered PPI allowed us to detect cancer driver pathways to highlight specific proteins as central hubs in these pathways. Simulating a systems approach concept, the presentation of cancer pathways could better guide research questions to focus on developing pathway blockers on highly convergent targets in the proteome. Systems approaches have emerged as a new way of thinking in cancer‐targeting efforts, mainly in response to the accumulation of setbacks in the traditional approach of single versus combinatorial target therapy. This new approach is already being employed in blood cancer biology in association with chromatin remodelling by oncogenic STAT3/5 action.^45^

#### 3.2.1 *Lung* *cancer*

Lung cancer is divided into two main histopathological types: small‐cell lung cancer and non‐small‐cell lung cancer. Small‐cell lung cancer arises from neuroendocrine cells and accounts for up to 15% of lung cancers. Most lung cancers are non‐small‐cell lung cancers (NSCLC), which are further divided into: adenocarcinoma, squamous cell carcinoma and large‐cell carcinoma. Adenocarcinoma is stratified by different driver mutations such as activating mutations in *K*‐*RAS* and *EGFR* and arises from alveoli‐lining epithelial cells of the airways. The STAT lung cancer interactome (Figure 4A) focuses predominantly on adenocarcinomas, which accounts for up to 90% of lung cancers.^46^ In the lung cancer interactome, we observe a larger number of central proteins aside from EGFR and STAT3. All these key proteins are actively involved in lung cancer. STAT3 and EGFR share a similar level of centrality in lung cancers, much more so than in the global interactome (Figure S1). This suggests a role for EGFR that is more important in the disease phenotype than in healthy lung tissue and is critical to lung cancer pathology. Furthermore, NSCLC pathway (Figure 4A) appears to be centralized around STAT3 and its direct interaction partners, alongside EGFR. The majority of lung cancer studies described STAT3 as an oncogene particularly with hyperactive EGFR signalling of autocrine IL‐6 stimulation; however, in the context of KRAS mutations, STAT3 possesses tumour suppressive activity. Such complications make targeting of STAT3 in NSCLC a difficult decision.^47^ While there are many proteins which act as partial communication hubs due to their centrality such as STAT1, ESR1 or TP53, the concentration of the lung cancer pathway around STAT3 and EGFR suggests that their inhibition would have the largest detrimental effect on the disease by preventing activation of drug resistance pathways upon combinatorial treatments. Targeting of oncogenic RAS or tumour suppressive/oncogenic TP53 mutations is challenging and will require innovative therapy concepts; however, as suggested by the interactome, the nuclear hormone receptor (e.g. ESR1) pathways in lung cancer also appear to be central (represented by the yellow hollows, Figure 4A) and can serve as a new viable target in these cancers. Future studies could evaluate anti‐estrogen drugs such as tamoxifen as part of combinatorial treatment approaches.

#### 3.2.2 *Breast* *cancer*

There are many subtypes of breast cancers initiating in different regions of the breast, such as the ductal areas, lobules or in more rare cases, within the connecting cell types. Triple‐negative breast cancer (TNBC) is the most aggressive and difficult to treat subtype, accounting for 15%–20% of breast cancers and is de novo resistant to estrogen therapy. Breast cancer biomarkers in TNBC include the Estrogen receptor (ER), Progesterone receptor (PR) and human epidermal growth factor receptor 2 (HER2) with all three being absent in TNBC. The World Health Organization (WHO) distinguished at least 18 different histological breast cancer types.^48^ Luminal breast cancers are the largest group, and they display the estrogen receptor (ER) as a biomarker of good prognosis if highly expressed. ER‐positive carcinomas account for ~70% of all cases of breast cancers in Western societies.^49^, ^50^ Whereas 50% of ER positive breast cancers displays PR expression as a second biomarker, HER2 is the third biomarker in breast cancer accounting for 15%–25% of cases. HER2 amplification is associated with metastasis, and its persistent activation promotes STAT3/5 oncoprotein activation; however, their role as being an oncogene or tumour suppressor protein in breast cancer is controversial due to complexity of the 18 different breast cancer subtypes or driver mutation context. Thus, biomarkers are highly relevant for the different breast cancer subtypes. Due to their heterogeneous driver mutation landscapes, treatment protocols involving personalized precision medicine have been described to be useful and effective.^51^ Because of tumour heterogeneity, different breast cancer subtypes are not accounted for, and subtype overlaps cannot be avoided in our network analysis of breast cancer. Nonetheless, analysis of simplified (or more homogenous) breast cancers (mainly luminal as opposed to the most aggressive TNBC) revealed that member‐exclusive STAT activity becomes more apparent when STAT PPIs are filtered in relation to STAT‐linked cancers.

In breast cancer, STAT3 has a large number of interactions and many of these interactions involve moderately central proteins in the STAT‐breast cancer interactome (Figure 3A). STAT3 is the most central protein, followed closely by EGFR, revealing a surprising similarity to lung cancer. This may in part explain why breast cancer might favour lung cancer metastasis, an insight that could obtain attention in the different breast cancer subtypes with or without lung metastasis. Out of all STATs, STAT3 and STAT1 possess the largest number of interactions and a large centrality governing communication between other proteins, being the most important STATs in the breast cancer network. Although the non‐STAT proteins such as TP53, ESR1, SRC and JAK2 are often linked to breast cancer, their dominance in the network is overshadowed by exclusion of non‐STAT interacting proteins. A general comparison of PPIs in diseased and healthy mammalian tissue shows that these proteins become more central to communication in the disease context highlighting upregulated protein–protein communication in breast cancer.^52^, ^53^ Although inhibition of STAT3 is considered to severely hinder the viability of breast cancer cells,^54^ this high degree of centrality and signalling redundancy between multiple central oncogenes again suggests that combination therapies targeting STAT3, and other key proteins including TP53, ESR1, EGFR or STAT1 may present with a higher degree of success in the clinic.^55^, ^56^

Signal transducer and activator of transcription interactome analysis of TNBC was performed by investigating tumour‐relevant pathways in this most aggressive highly metastasising subtype and by isolating the specific proteins. Compared with the overall breast cancer interactome (mainly comprising of the luminal subtype), STAT influence on the TNBC interactome is predominantly shifted towards STAT1 and STAT3 (Figure 3B). However, all of the central non‐STAT proteins (TP53, EGFR and ESR1) in the breast cancer network are still interacting and they are central to the TNBC interactome. SRC and JAK2 kinases also display a more distinct influence in TNBC. The centrality of ESR1 in TNBC is again interesting as its epigenetic silencing has been observed in TNBC patients from India.^57^ These findings suggest that while several STATs collectively govern breast cancer, STAT1 and STAT3 become more influential proteins to TNBC progression.

#### 3.2.3 *Prostate* *cancer*

Prostate cancers are androgen‐driven tumours, and they are classified as luminal adenocarcinomas in the majority of cases.^58^ Luminal adenocarcinomas are castration‐sensitive (androgen‐responsive) upon anti‐androgen treatment. The other types of prostate cancers are neuroendocrine prostate cancer or small‐cell carcinoma, which account for less than 5%, and these are not covered by our analysis. In prostate luminal adenocarcinomas, we observe that STAT1/3/5A/5B are all interacting with a substantial number of proteins with moderate centrality (Figure 3C). STAT6 is also present although it has significantly fewer interactions with low centrality suggesting it is not a critical protein. STAT3 interacts with the largest number of proteins in a diverse array of gene ontologies and many of the shared interaction partners in the centre of the network. STAT3 is known to be constitutively activated in prostate tumours and related cell lines.^59^ Again, similar to our observations in lung cancer or breast cancer, a key STAT3 interaction partner is an ERBB family member, namely EGFR, the second most central protein in the network (Figure 3C). The STAT interactome in prostate cancer supports that EGFR is a key signal transducer. Indeed, EGFR is already known for promoting the motility and growth of prostate cancer cells.^60^, ^61^ The interactions passing through the network are centralized on STAT3 and EGFR, with STAT1/5A/B, TP53 and ESR1 playing a supporting role.^62^, ^63^ Interestingly, both STAT3 and STAT5A interact with STAT1 and STAT5B but not with each other. EGFR, however, interacts with all STATs except STAT6. This appears to be the cause of the substantial centrality of EGFR as it interacts with all the major STATs present in prostate cancer. This also illuminates a divergence between STAT3 and STAT5A, and this is bridged by other STATs and EGFR interaction.

#### 3.2.4 *Colorectal* *cancer*

In contrast to prostate cancer, however, the non‐STAT proteins appear to be more important in the communication between STATs in colorectal cancer (Figure 4B). Although STAT5A (15 interactions) and STAT6 (8 interactions) are interconnected, STAT1 (32 interactions) and STAT3 (45 interactions) have more interactions and show higher centrality (Figure 4B). Collectively, these findings suggest that among the STATs, STAT1 and STAT3 are by far the most important in colorectal cancer progression while other STATs such as STAT5A, STAT5B and STAT6 are vastly understudied.^64^, ^65^ EGFR, TP53 and ESR1 also have high centrality and degree in colorectal cancers. TP53 and ESR1 are both more central to the interactome than STAT1 and STAT5A, their respective exclusive interaction partners, both of which have been shown to be linked to colorectal cancer.^66^ This situation arose because a larger number of TP53 and ESR1 interactions have been reported in association with colorectal cancer, while many of the physiological interactions of STAT1 and STAT5A in healthy cells were not identified in colorectal cancer. Notably, STAT1 is well known to interact with TP53 and both have been shown to collaborate in mediating the apoptosis response in colorectal cancer.^67^

#### 3.2.5 *Liver* *cancer*

Liver cancer shows one of the most surprising interactomes where the non‐STAT proteins have a larger role than even STAT3 (Figure 4C). TP53 has by far the largest centrality of any protein shown here followed closely by EGFR. Connecting these two central liver cancer proteins are STAT1 and ESR1. These four proteins have the largest influence in the STAT interactome in liver cancer with STAT3 following closely behind. STAT1, EGFR, ESR1 and TP53 have all been shown to be important for the progression of liver cancer, along with the STAT3 signalling pathway.^68^, ^69^, ^70^, ^71^, ^72^, ^73^, ^74^ Notably, two clinical trials, a phase III trial on tamoxifen (an ESR1 antagonist) and a phase II trial of tamoxifen in combination with cisplatin and doxorubicin hydrochloride, have already been performed for the treatment of liver cancers.^75^, ^76^ The postulated central role of the estrogen receptor should be further investigated to address whether both classes of estrogen receptors (ERα and ERβ) are involved and relevant in hepatocellular carcinoma progression.

There is a dominant role for the IL‐6‐JAK‐STAT component reported in many liver cancer studies, which clearly implicates the JAK1/JAK2‐STAT3 axis as oncoproteins,^77^ but expression of JAK kinases in general is much lower than the expression of STAT proteins, causing a bias in systems biology analysis. However, JAK kinases are frequently mutated, and if a gain or loss of function mutation would be found, then this analysis would strongly influence the systems biology insight. This can be best illustrated for the most abundant and frequent JAK mutation, which is found in JAK2V617F, driving myeloproliferative neoplasms (MPN). However, we did not include MPN in our blood cancer analysis since we wanted only to include acute leukaemia or lymphoma insights. The dominant role of JAK2 in MPN is described elsewhere, and we have not included these in our analysis of blood cancers that are discussed next due to the large diversity of hematopoietic cancers.^78^

#### 3.2.6 *Blood* *cancers*

Hematopoietic cancers are heterogeneous diseases of mesenchymal origin. The central role of perturbed JAK/STAT signalling in the initiation and progression of blood cancers is not surprising given its essential node in normal haematopoiesis—the development and function of blood cells of myeloid and lymphoid lineages. There, it is involved in the transmission of complex signalling pathways that are mediated by numerous cytokines and their receptors.^79^

Myeloid STAT signalling is distinct from lymphoid STAT networks. In the myeloid branch, binding of cytokines to their receptors initiates JAK activation, which in turn ignites particular STAT proteins, MAPK and PI3K‐AKT‐mTOR pathway in a receptor‐specific fashion. In our analysis, EGFR and TP53 are the only central non‐STATs in the two networks of hematopoietic cancers (Figure 5A, B) while ESR1 is central in only myeloid leukaemia. EGFR displays a comparable level of centrality to STATs, and as such, combination therapies targeting EGFR have been proven effective in AML cells. The centrality of TP53, a well‐known interactor of STAT1/3/5 proteins, is the most important tumour suppressor transcription factor in human cancer with half the cases carrying a genetic mutation or loss. Quite interestingly, TP53 represses STAT5 transcription, but mutated TP53 is shown to boost STAT5 transcription in AML cells.^31^ On the contrary, lymphoid JAK‐STAT signalling, cytokines that bind to common‐γ‐chain containing receptors, such as IL‐2/4/7/9/15/21, are central in physiological T‐, NK‐ and B‐cell development and function.^80^, ^81^ Hyperactivation of JAK1/JAK3 results in high, oncogenic STAT3 and STAT5 activity.^82^, ^83^

Overall, STAT1/3/5A/5B are distinctly activated and/or overexpressed in hematologic neoplasms. STAT1 acts as a leukaemogenic oncogene in B‐lymphoid leukaemia, and *STAT3* mutations in the SH2 domain (affecting dimerization and activation) have been detected in numerous leukaemia samples in patients with LGL.^84^, ^85^, ^86^ Whereas mutations in *TP53* are associated with poor survival in AML patients, ESR1 methylation is reported to correlate with hypermethylation of several oncogenes linked to AML^87^, ^88^ and more work is needed to understand centrality of EGFR, TP53 and ESR1 in blood cancers. In conclusion, STAT1 and STAT3 are the only STATs interacting with the majority of proteins in the hematopoietic cancer interactome network, showing a high degree of centrality (Figure 5, Figure S4). Comparably, the other STATs (STAT2, STAT4 and STAT6) display a smaller number of interactions. This is unsurprising due to the specific expression pattern of STAT4 and STAT6, or a more restricted role of STAT2 in viral/bacterial defence.

### 3.3 Subcellular localization of STAT proteins

Due to their common function of transferring signals from the cytoplasm to the nucleus, all STAT members appear in both the cytoplasm and the nucleus and they interact with many of the same proteins (Figure S3). Since the cytoplasm and nucleus is where the majority of the STAT pathway‐associated proteins reside (see Figure 1), interactomes filtering these two subcellular compartments are very similar compared with the full STAT interactome. In the cytoplasmic STAT interactome, given their high centrality, STAT3, EGFR and STAT1 are responsible for the bulk of communication between proteins (Figure S3A). In the nucleus, however, TP53, STAT5A and ESR1 appear to be central communication hubs (Figure S3B). STAT3 and STAT5 play a regulatory role in the nucleus.^89^ Taken together, these findings and associations suggest that the transmission of information is more delocalized in the nucleus than in the cytoplasm. Thus, for therapeutic strategies, targeting central cytoplasmic proteins would provide less room for the oncogenic signalling to be offloaded to another protein.

STAT3 delocalizes to all four subcellular compartments: the nucleus, cytoplasm, mitochondrion and plasma membrane, and it interacts with SRC, Receptor of activated protein C kinase 1 (RACK1), MAPK1/3, tyrosine‐protein kinase Lyn (LYN), mitochondrial GRIM1 and Sorcin (SRI) across these compartments. Mitochondrial STAT3 action is required for RAS‐RAF transformation and is triggered via actions such as the stress kinase‐mediated serine phosphorylation of STAT3.^90^, ^91^ Apart from STAT3, both STAT1 and STAT5 are also found to translocate to mitochondria (Figure S3C). Unlike the nucleus and cytoplasm, one of the direct interaction partners of STAT3 in the mitochondria was described to be the proteins of complex I and complex III of the respiratory chain and 2‐Oxoglutarate dehydrogenase (OGDHL), which is observed in our interactomes and has a high degree of centrality. There are two other highly central proteins in the mitochondria, TP53 and Dihydrolipoyl dehydrogenase (DLD). DLD and OGDHL both function as oxidoreductases, and OGDHL is associated with cervical cancer.^92^ The relatively large numbers of central proteins in this network suggests that STAT3 is not as important to mitochondrial signalling as cytosolic or nuclear localisation of STAT3. Recently, STAT3 has been shown to be located within a portion of the ER associated with the mitochondria instead of the mitochondria itself, putting its crucial role within the mitochondria into question.^93^ Furthermore, the presence of multiple proteins in the above with similar functions (i.e., oxidoreductases) in the mitochondrion indicates the existence of a redundant molecular pathway. In the plasma membrane, STAT3, along with EGFR, dominates the network, with a similar number of interactions and degree of centrality (Figure S3D). Due to a high number of shared interactions in the plasma membrane interactome, both STAT3 and EGFR appear critical for signalling across the plasma membrane, followed consecutively by JAK1/2 and SRC. Due to its servitude as a reliable interaction partner and communication hub for STATs from the plasma membrane to the nucleus, EGFR disruption can clearly be foreseen and is shown to be beneficial in arresting or eradicating cancer cell growth.^94^ Notably, STAT2 is the only other STAT delocalized to the plasma membrane although it does not appear to play a key role in the communication there. Overall, while the interactomes of the nucleus and cytoplasm appear remarkably similar to the whole STAT interactome, STAT3 appears to be the only influential STAT in the plasma membrane and displays minimal importance in the mitochondria.

## 4 FUTURE DIRECTIONS AND CONCLUSIONS

In all the carcinoma and hematopoietic cancer networks, STAT1 and STAT3 interact with a similar panel of proteins, including MTOR, SRC and EGFR (Figure 3, Figure 4, Figure 5 and Figure S4). The observed crosstalk between these STATs as well as between STAT5A and STAT5B suggests that the formation of a heterodimer is important in diseased states. Interestingly, although a STAT1‐STAT5A interaction is reported in breast and lung cancers or leukaemia, there is no evidence for a STAT1‐STAT5B or a STAT3‐STAT5A interaction observed in any of the interactomes investigated. While all STATs interact with a larger number of oncogenes than tumour suppressors, the ratio of oncogenes to tumour suppressors is highest for STAT5A and STAT5B (Figure S5). For each disease investigated in this paper, a STAT1‐STAT3 interaction has been consistently reported (Figure 3, Figure 4, Figure 5 and Figure S4). These interactions clearly indicate the importance of STAT crosstalk in diseased states, and hence, their ongoing exploration in drug discovery efforts will be important. Targeting of STATs is a long‐standing strategy, and a variety of small‐molecule drugs and degraders such as fludarabine, pimozide, sulforaphane, pyrimethamine and SD‐36 have been developed. Our analysis indicates that although inhibition of STATs is said to severely hinder the viability of certain cancers, combinatorial therapy of STATs alongside other key proteins including TP53, ESR1 and EGFR may result in a higher degree of success especially in solid cancers. Furthermore, based on our interactome, ESR1 appears to be central and it can serve as a new viable combinatorial target in lung cancers. In blood cancers, however, STAT1, STAT3 and STAT5 play a dominant role and single STAT‐targeting therapies may suffice, also reflecting their less complex genetic driver mutation landscape. In most cases of STAT‐linked diseases such as solid carcinomas, a combination therapy of STAT3/5 dual inhibitors or combination therapy with upstream TKI such as ERBB family member blockers or JAKinibs will likely be more successful.

## CONFLICT OF INTEREST

The authors declare no competing interests.

## AUTHOR CONTRIBUTIONS

**Fettah Erdogan:** Data curation (equal); Formal analysis (equal); Investigation (equal); Methodology (equal); Writing – original draft (lead); Writing – review & editing (lead). **Tudor Bogdan Radu:** Data curation (lead); Formal analysis (equal); Methodology (supporting); Software (lead); Visualization (lead); Writing – original draft (supporting). **Anna Orlova:** Visualization (supporting); Writing – review & editing (supporting). **Abdul Khawazak Qadree:** Visualization (supporting); Writing – review & editing (supporting). **Elvin Dominic de Araujo:** Project administration (supporting); Resources (supporting); Writing – review & editing (supporting). **Johan Israelian:** Investigation (supporting). **Peter Valent:** Writing – review & editing (supporting). **Satu Mustjoki:** Writing – review & editing (supporting). **Marco Herling:** Writing – original draft (supporting); Writing – review & editing (supporting). **Richard Moriggl:** Supervision (equal); Writing – review & editing (supporting). **Patrick Thomas Gunning:** Supervision (equal).

## Supporting information

Figure S1

Figure S2

Figure S3

Figure S4

Figure S5

Table S1

Supplementary Material
