# Supplementary figures and images for: JAK‐STAT core cancer pathway: An integrative cancer interactome analysis
Source: J Cell Mol Med. 2022 Mar 1;26(7):2049–62. doi: 10.1111/jcmm.17228 (PMC8980946; doi:10.1111/jcmm.17228)

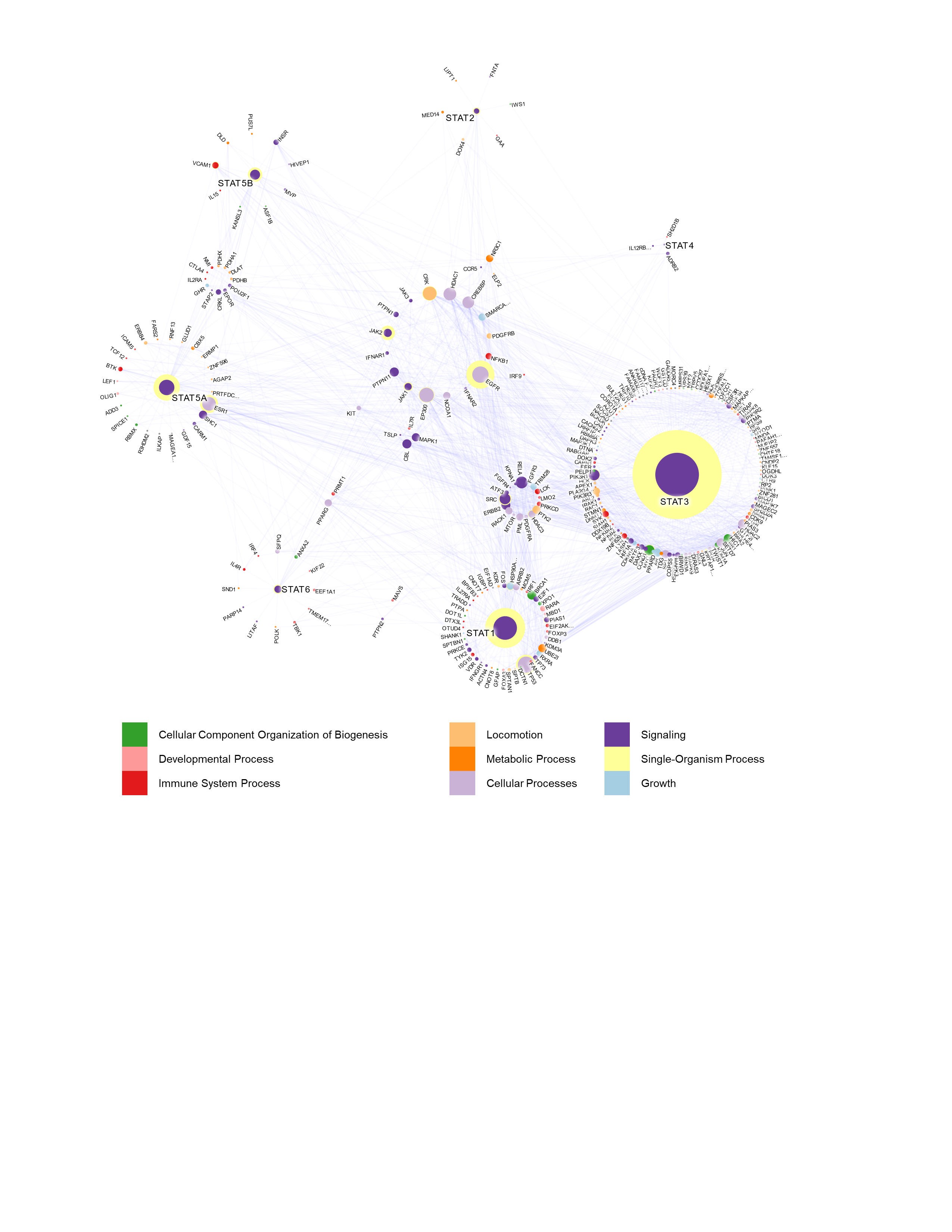

Supplement: Supplementary file 1 — Figure S1 [file JCMM-26-2049-s002.png]

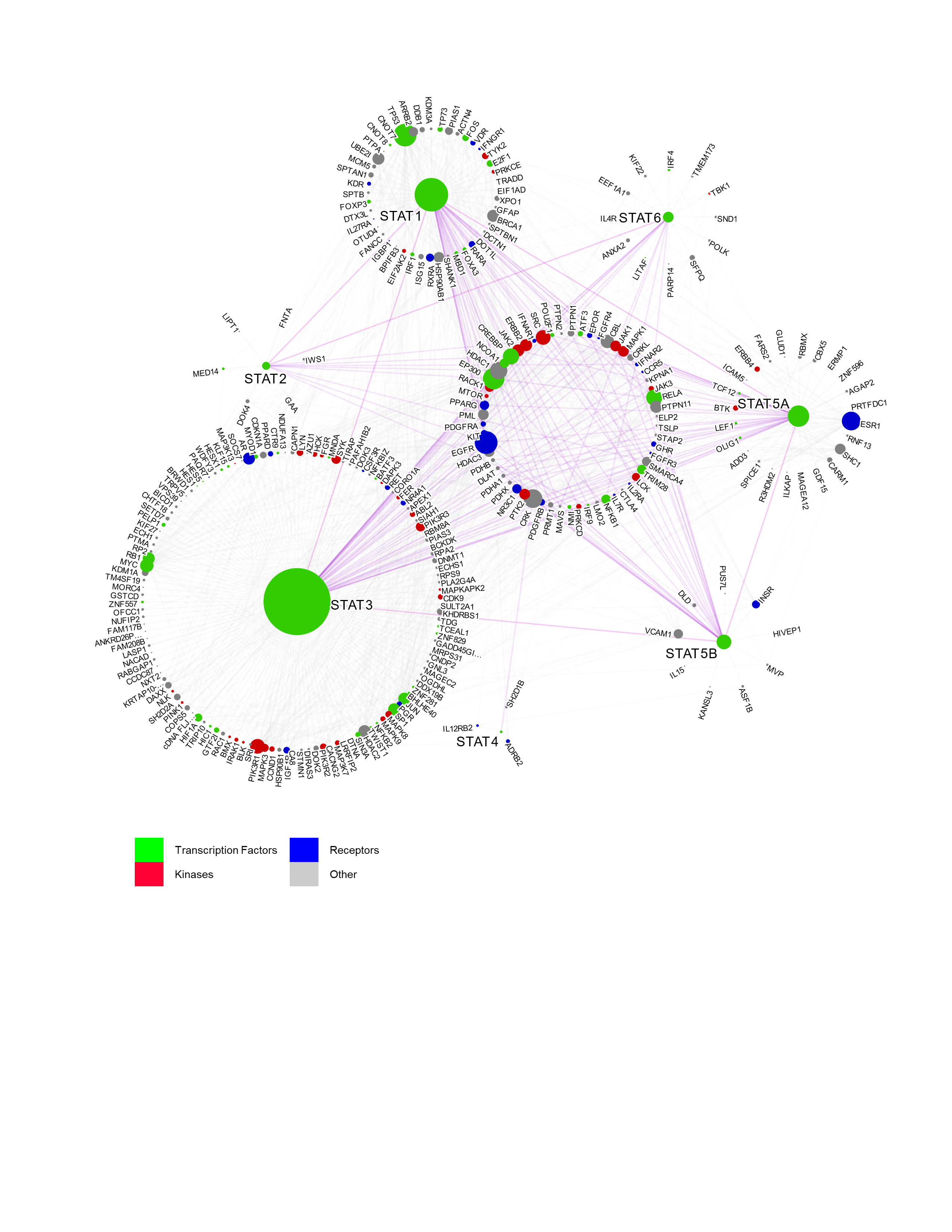

Supplement: Supplementary file 2 — Figure S2 [file JCMM-26-2049-s007.png]

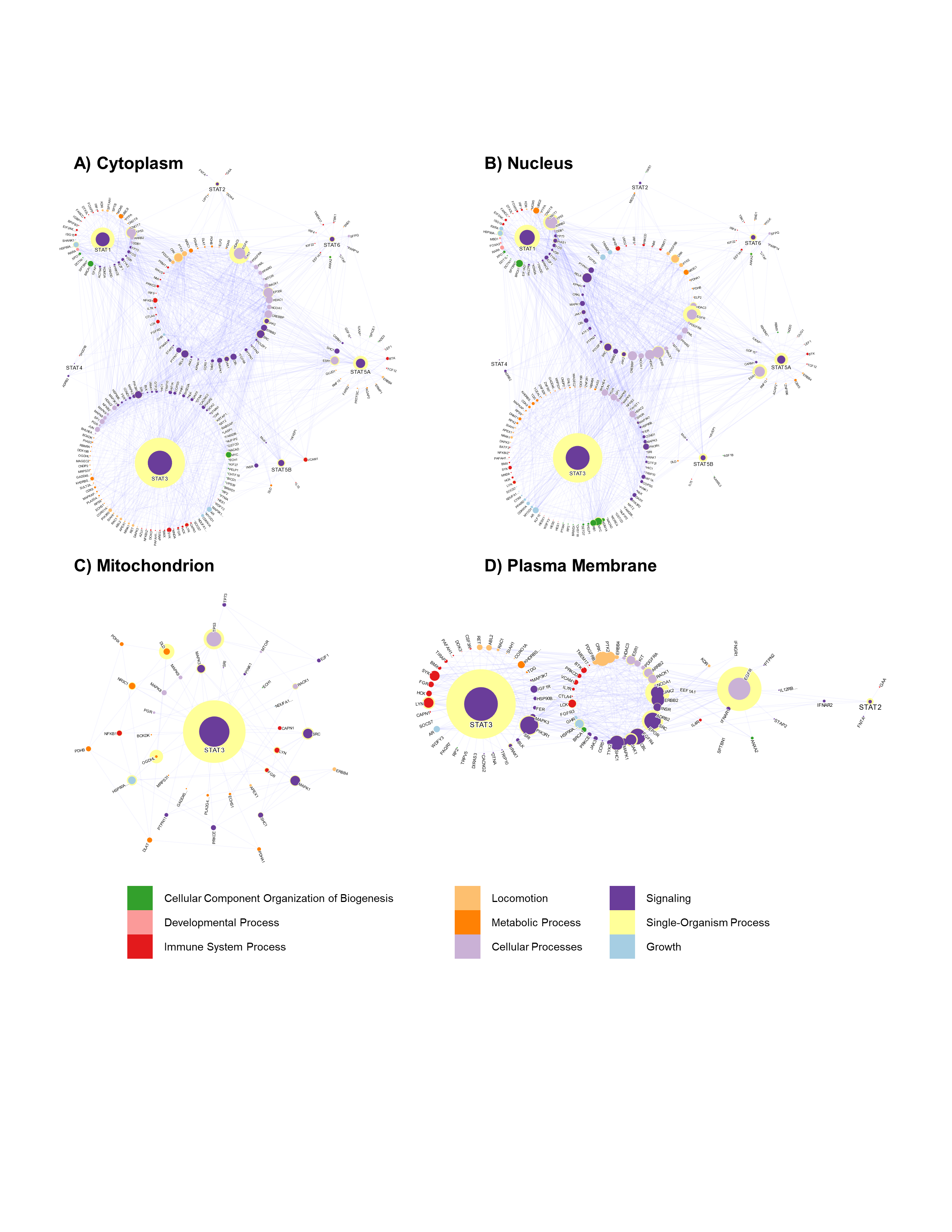

Supplement: Supplementary file 3 — Figure S3 [file JCMM-26-2049-s003.png]

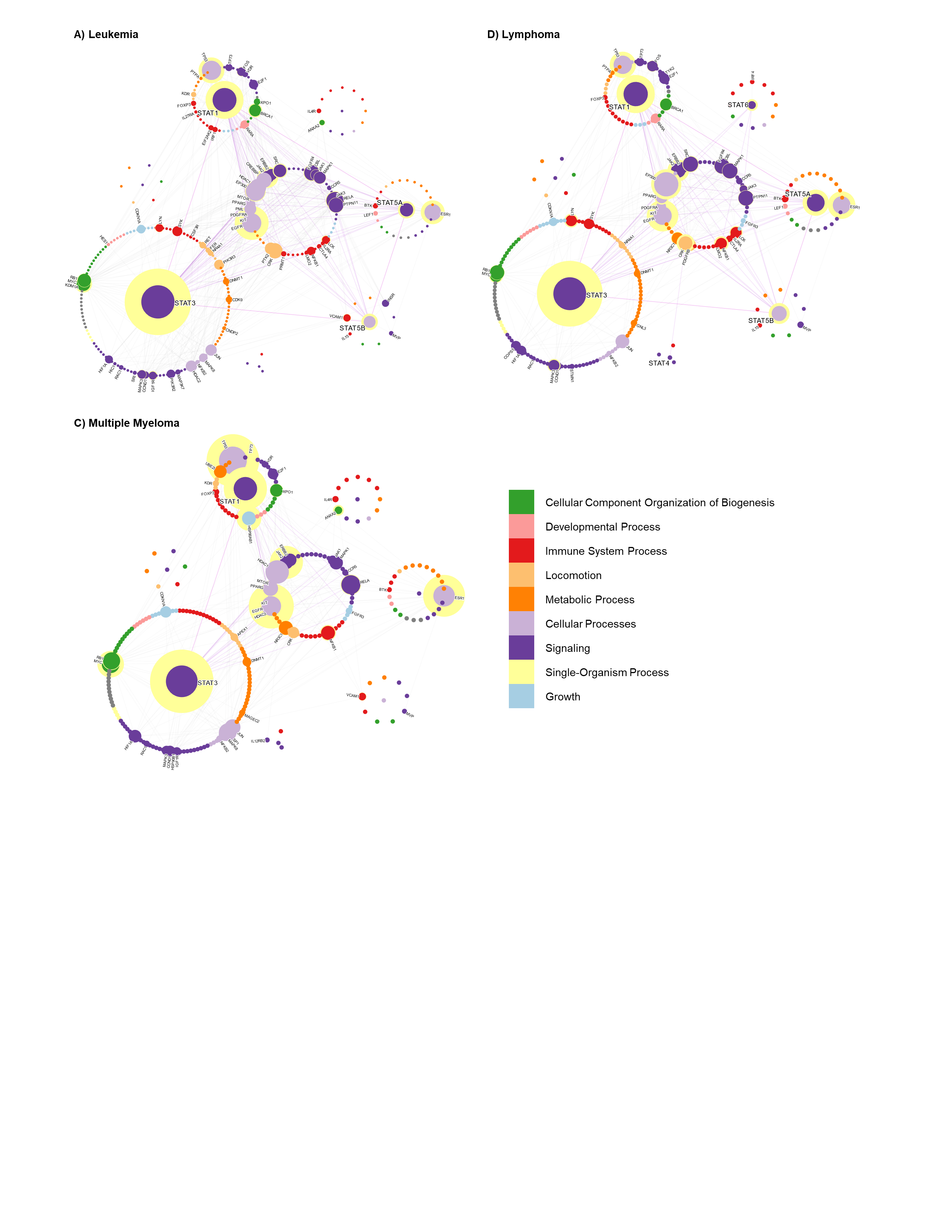

Supplement: Supplementary file 4 — Figure S4 [file JCMM-26-2049-s006.png]

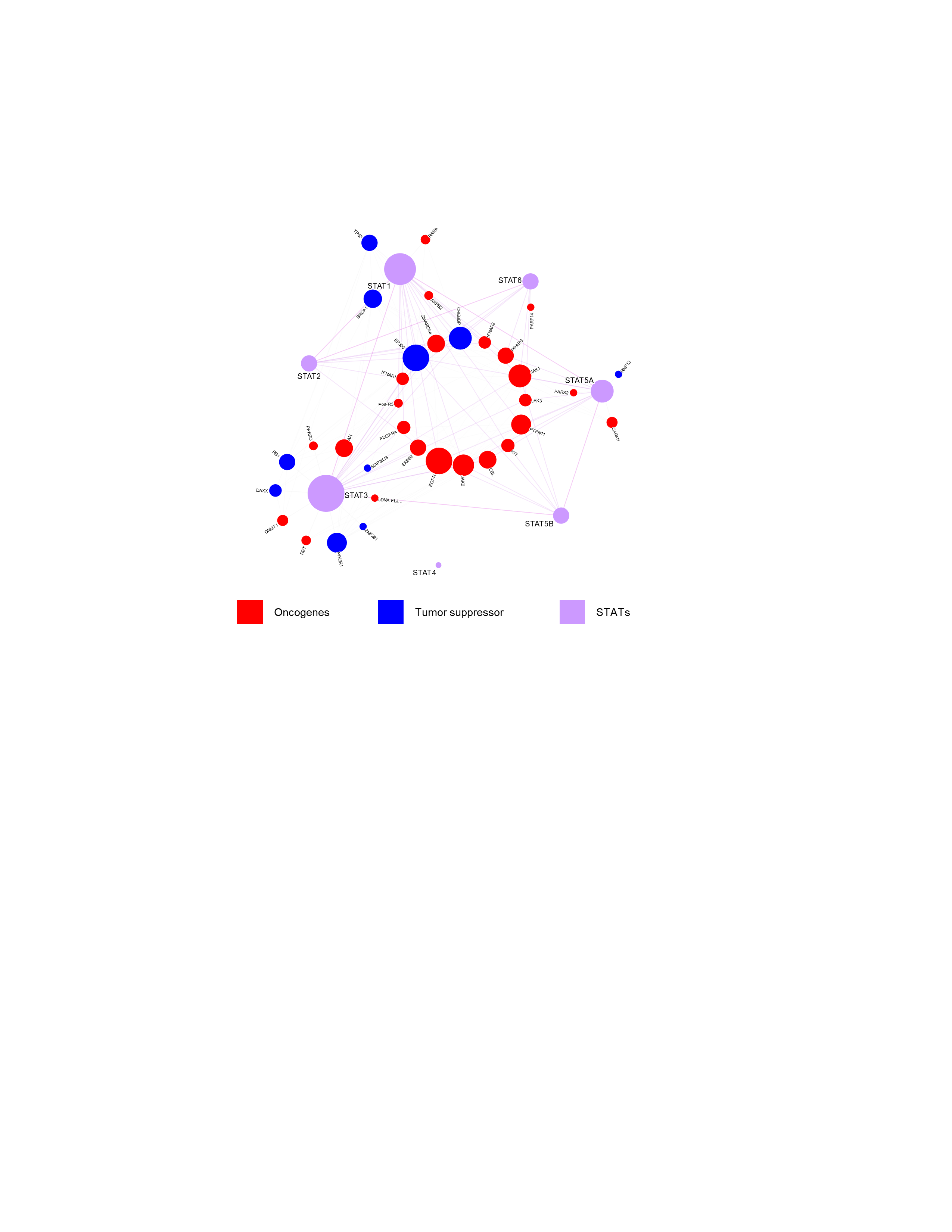

Supplement: Supplementary file 5 — Figure S5 [file JCMM-26-2049-s004.png]
