# Supplementary material for: JAK‐STAT core cancer pathway: An integrative cancer interactome analysis
Source: J Cell Mol Med. 2022 Mar 1;26(7):2049–62. doi: 10.1111/jcmm.17228 (PMC8980946; doi:10.1111/jcmm.17228)
Supplement: Supplementary file 6 — Table S1 [file JCMM-26-2049-s001.pdf]

STAT UniProt	PPI UniProt ID	STAT	PPIs	Pubmed IDs
P42224	Q00597	STAT1	FANCC	10848598;11520787;14499622;21836163;25402006
P42224	Q8TDB6	STAT1	DTX3L	24886089;26479788
P42224	Q7Z434	STAT1	MAVS	21903422
P42224	P52294	STAT1	KPNA1	10964507;12048190;12740372;16298512;21836163;25402006
P42224	Q8N9N8	STAT1	EIF1AD	16189514;25416956
P42224	Q02156	STAT1	PRKCE	20353823;24825907;25402006
P42224	Q6UWB1	STAT1	IL27RA	12734330;21836163
P42224	Q92793	STAT1	CREBBP	10848577;15695802;15894584;16481475;19915063;25402006;25451029
P42224	Q9UIS9	STAT1	MBD1	19074829;25402006
P42224	Q13547	STAT1	HDAC1	14645718;16481475;25402006
P42224	Q13263	STAT1	TRIM28	18037959;18381204
P42224	Q01804	STAT1	OTUD4	25416956
P42224	P42345	STAT1	MTOR	12807916;19553685;25241761;25402006
P42224	Q05397	STAT1	PTK2	11278462;20576130;25241761;25402006;doi:10.1007/978-3-540-73060-
P42224	Q9Y566	STAT1	SHANK1	20936779
P42224	Q04206	STAT1	RELA	16481475;23023127;25241761;25402006
P42224	Q00978	STAT1	IRF9	16082366;17923090;24065129;25402006;8943351;9242679;doi:10.1007/
P42224	Q06124	STAT1	PTPN11	12270932;18832710;21836163;25402006;27229929
P42224	Q05655	STAT1	PRKCD	11839738;11972023;12637327;12807916;12817007;14963018;15322115;19553685;21836163;25402006;7543024;7690989;9355737
P42224	Q13813	STAT1	SPTAN1	20936779;25402006
P42224	Q14203	STAT1	DCTN1	20936779
P42224	P63244	STAT1	RACK1	11301323;12960323;25402006;doi:10.1007/978-3-540-73060-6_4
P42224	P63279	STAT1	UBE2I	12356736;12764129;14517261;25402006
P42224	Q9UFF9	STAT1	CNOT8	23386060
P42224	Q09472	STAT1	EP300	10464260;11134049;11923478;12403783;15824515;18566411;19915063;25402006;26479788;26504087;8986769;9843502;doi:10.1007/978-3-
P42224	P78318	STAT1	IGBP1	19553685;20936779
P42224	Q16531	STAT1	DDB1	21988832
P42224	Q99873	STAT1	PRMT1	11257227;12171910;21836163;25402006
P42224	Q01082	STAT1	SPTBN1	20936779
P42224	Q9UIV1	STAT1	CNOT7	23386060
P42224	Q15257	STAT1	PTPA	19553685

P42224	Q9Y4C1	STAT1	KDM3A	25535969
P42224	P42229	STAT1	STAT5A	10358045;16082366;21836163;23023127;25241761;25402006;doi:10.10
P42224	Q9BZS1	STAT1	FOXP3	19124747
P42224	P55318	STAT1	FOXA3	25609649
P42224	Q01094	STAT1	E2F1	20195357;25402006
P42224	P59826	STAT1	BPIFB3	17353931
P42224	P52630	STAT1	STAT2	10446176;10490982;12048190;14722125;15825084;15978943;16082366;17923090;21178011;21268015;21836163;23023127;24065129;2540200
P42224	Q15628	STAT1	TRADD	10848577;21836163;doi:10.1007/978-3-540-73060-6_4
P42224	P42224	STAT1	STAT1	12923054;14704793;15284440;15780933;16007122;18591661;21914072
P42224	P48551	STAT1	IFNAR2	11301323;21836163;25402006;9121453;doi:10.1007/978-3-540-73060-
P42224	Q8TEK3	STAT1	DOT1L	22002246
P42224	P51532	STAT1	SMARCA4	16195385;20353823;21079652;25402006
P42224	P22607	STAT1	FGFR3	10918587;11294897;11839738;12637327;12817007;15322115;21836163
P42224	P11277	STAT1	SPTB	25814554
P42224	O15379	STAT1	HDAC3	16481475;25402006
P42224	P06239	STAT1	LCK	10918587;11294897;11839738;12637327;12817007;15322115;25402006;7543024;7657660;7690989;doi:10.1007/978-3-540-73060-6_4
P42224	P37231	STAT1	PPARG	25402006;26504087
P42224	P25791	STAT1	LMO2	16189514;25416956
P42224	P33992	STAT1	MCM5	11248027;25402006;9843502;doi:10.1007/978-3-540-73060-6_4
P42224	P29597	STAT1	TYK2	10918587;11294897;11839738;12637327;12817007;12960323;15322115;21836163;25402006;7543024;7657660;7690989;doi:10.1007/978-3-540-
P42224	P18847	STAT1	ATF3	19647793;25402006
P42224	P01100	STAT1	FOS	12788789;21836163;25241761;25402006;8662591
P42224	O14980	STAT1	XPO1	22833565;25402006;26673895;9205132;doi:10.1007/978-3-540-73060-
P42224	P12931	STAT1	SRC	10358079;14978237;21836163;25402006;9344858
P42224	P22455	STAT1	FGFR4	10918587;11294897;11839738;12637327;12817007;15322115;25402006;7543024;7657660;7690989;doi:10.1007/978-3-540-73060-6_4
P42224	O43707	STAT1	ACTN4	20936779
P42224	P05161	STAT1	ISG15	16009940;16139798;25402006
P42224	P09619	STAT1	PDGFRB	23023127;23397142;25402006;8549654;9484840
P42224	P08238	STAT1	HSP90AB1	20936779
P42224	P04626	STAT1	ERBB2	16273093;25241761;25402006

P42224	P10276	STAT1	RARA	25303530
P42224	P23458	STAT1	JAK1	10918587;10982844;11294897;11722592;11839738;12637327;12817007;14978237;15284024;15322115;15978943;25402006;7543024;7657660;7
P42224	P19793	STAT1	RXRA	21988832;25402006
P42224	P11473	STAT1	VDR	11909970;25402006;doi:10.1007/978-3-540-73060-6_4
P42224	P29590	STAT1	PML	25402006;25733689;25812002
P42224	P10721	STAT1	KIT	10358045;25241761;25402006;9355737
P42224	P14136	STAT1	GFAP	20936779
P42224	P19525	STAT1	EIF2AK2	11278865;21836163;25402006;9135145;doi:10.1007/978-3-540-73060-
P42224	P04637	STAT1	TP53	14602726;15217838;17346710;17992189;23023127;25402006
P42224	O60674	STAT1	JAK2	10918587;11152457;11294897;11839738;12637327;12817007;14978237;15284024;15322115;16082366;19834108;20353823;21836163;2425595
P42224	P35968	STAT1	KDR	10961983;15703780;25402006
P42224	O75925	STAT1	PIAS1	10805787;11257227;12171910;12855578;16082366;17371985;18566411;19136629;21836163;25241761;25402006;9724754;doi:10.1007/978-3-
P42224	P32121	STAT1	ARRB2	17620599
P42224	P10914	STAT1	IRF1	10764778;16082366;16512786;17525742;19124747;24012417;25402006
P42224	P17706	STAT1	PTPN2	12171910;12923054;19171783;21836163;25402006;doi:10.1007/978-3-
P42224	P00533	STAT1	EGFR	10358079;10918587;11294897;11839738;12070153;12637327;12817007;15284024;15322115;16273093;21836163;23956138;24658140;2479726
P42224	P15260	STAT1	IFNGR1	10848598;15494521;15780933;21689637;21836163;25402006;7514165;8156998;8605876;8662591;8910398;doi:10.1007/978-3-540-73060-6_4
P42224	P16234	STAT1	PDGFRA	25241761;25402006;9484840
P42224	O15350	STAT1	TP73	17346710;25402006
P42224	P38398	STAT1	BRCA1	10792030;25402006;doi:10.1007/978-3-540-73060-6_4
P52630	Q92793	STAT2	CREBBP	10464260;25402006;28514442;8848048
P52630	Q8TEW6	STAT2	DOK4	25814554
P52630	Q96ST2	STAT2	IWS1	21988832
P52630	Q9Y234	STAT2	LIPT1	26186194;28514442
P52630	Q13547	STAT2	HDAC1	14645718;25402006
P52630	Q09472	STAT2	EP300	10464260;15154850;18678383;21836163;25402006;doi:10.1007/978-3-
P52630	Q00978	STAT2	IRF9	15978943;17923090;21178011;21268015;21836163;21903422;24065129;25402006;28514442;8943351;9242679;doi:10.1007/978-3-540-73060-
P52630	P52630	STAT2	STAT2	8605877

P52630	O60244	STAT2	MED14	12509459
P52630	P00533	STAT2	EGFR	24658140;25402006
P52630	P17181	STAT2	IFNAR1	11786546;12220192;15356134;16082366;21836163;25402006;7559568;<br>8605876;9121453;9677371;doi:10.1007/978-3-540-73060-6_4
P52630	P10253	STAT2	GAA	21988832
P52630	P51532	STAT2	SMARCA4	12244326;25402006
P52630	P48551	STAT2	IFNAR2	11786546;12220192;17923090;18456457;21836163;25402006;9121453;
P52630	P49354	STAT2	FNTA	21988832
P40763	Q92665	STAT3	MRPS31	21988832
P40763	Q06520	STAT3	SULT2A1	21988832
P40763	Q15170	STAT3	TCEAL1	26186194;28514442
P40763	Q9NR55	STAT3	BATF3	25814554
P40763	Q9NSI6	STAT3	BRWD1	25814554
P40763	Q9Y5S9	STAT3	RBM8A	18503751;25402006
P40763	Q8IZS5	STAT3	OFCC1	25814554
P40763	P45983	STAT3	MAPK8	10446219;10521505;11350938;12576423;12763138;14551213;15979846<br>;21836163;25402006;9343414;9872331;doi:10.1007/978-3-540-73060-
P40763	Q92769	STAT3	HDAC2	15653507;25241761;25402006
P40763	P78347	STAT3	GTF2I	25402006;9584171
P40763	Q8IZL8	STAT3	PELP1	15994929;25402006
P40763	Q86VH2	STAT3	KIF27	25609649
P40763	Q9UBF1	STAT3	MAGEC2	27775077
P40763	Q05655	STAT3	PRKCD	10446219;10521505;11335711;11350938;12576423;12763138;14551213
P40763	Q6NSI1	STAT3	ANKRD26P1	25609649
P40763	Q96G01	STAT3	BICD1	21988832
P40763	P51681	STAT3	CCR5	11350939;14674010;25402006
P40763	Q9BXM7	STAT3	PINK1	25814554
P40763	Q9BVP2	STAT3	GNL3	21988832
P40763	Q92569	STAT3	PIK3R3	25402006;25814554
P40763	P51692	STAT3	STAT5B	16082366;21836163;23023127;25241761;25402006;9398404;doi:10.100
P40763	P43405	STAT3	SYK	10825200;20133729;21516116;25402006;25416956
P40763	Q6ZVU3	STAT3	cDNA FLJ42079 fis	25814554
P40763	Q9Y4J8	STAT3	DTNA	25814554
P40763	Q9NVE4	STAT3	CCDC87	25814554

P40763	P51451	STAT3	BLK	25402006;25416956
P40763	Q8N988	STAT3	ZNF557	25814554
P40763	Q13011	STAT3	ECH1	21988832
P40763	P42224	STAT3	STAT1	11594781;11722592;12070153;15284024;16007122;16082366;21836163;23023127;25241761;25402006;25609649;28514442;8662591;doi:10.100
P40763	P58753	STAT3	TIRAP	21903422
P40763	Q92793	STAT3	CREBBP	11239394;15649887;15653507;25402006
P40763	Q9Y698	STAT3	CACNG2	26186194;28514442
P40763	Q6P1L5	STAT3	FAM117B	25814554
P40763	Q6PD62	STAT3	CTR9	17911113
P40763	Q5VWN6	STAT3	FAM208B	25814554
P40763	P51532	STAT3	SMARCA4	15286705;25402006
P40763	Q969D9	STAT3	TSLP	10570284;11418668
P40763	P63244	STAT3	RACK1	16382134;25402006
P40763	Q9UBX0	STAT3	HESX1	25814554
P40763	Q7Z417	STAT3	NUFIP2	25814554
P40763	Q00653	STAT3	NFKB2	16651533;25402006;25771405
P40763	Q03181	STAT3	PPARD	25402006;25814554
P40763	Q86WK9	STAT3	PAQR7	25814554
P40763	P42345	STAT3	MTOR	10660304;15522880;23329839;25402006;doi:10.1007/978-3-540-73060-
P40763	Q96KP4	STAT3	CNDP2	25814554
P40763	Q8TAE8	STAT3	GADD45GIP1	18200042;25402006;25416956
P40763	P46781	STAT3	RPS9	21988832
P40763	Q9ULD0	STAT3	OGDHL	21988832
P40763	Q13569	STAT3	TDG	25814554
P40763	Q8IZQ1	STAT3	WDFY3	25814554
P40763	Q16665	STAT3	HIF1A	15735682;18985005;25402006
P40763	Q14469	STAT3	HES1	15156153
P40763	Q13263	STAT3	TRIM28	18037959
P40763	Q96ST3	STAT3	SIN3A	22783022
P40763	Q04206	STAT3	RELA	12057007;14593105;19345327;23335796;25402006;doi:10.1007/978-3-
P40763	P40763	STAT3	STAT3	14704793;15223310;15653507;16007122;17676033;20686606;21325026;21988832;23750211;25609649;7510216;9373245;9647732
P40763	P42684	STAT3	ABL2	25402006;25814554

P40763	Q9UBE8	STAT3	NLK	15764709;21836163;25402006
P40763	Q9UER7	STAT3	DAXX	16331268;25402006
P40763	Q05397	STAT3	PTK2	10925297;25241761;25402006;doi:10.1007/978-3-540-73060-6_4
P40763	Q14526	STAT3	HIC1	24067369;27085461
P40763	Q9BYH8	STAT3	NFKBIZ	19595668;21988832
P40763	Q9Y2X9	STAT3	ZNF281	25814554
P40763	Q9NP31	STAT3	SH2D2A	25402006;25814554
P40763	Q9UIH9	STAT3	KLF15	25814554
P40763	P45984	STAT3	MAPK9	20871632;25402006
P40763	Q8IUQ4	STAT3	SIAH1	21988832;27871173
P40763	P60409	STAT3	KRTAP10-7	25814554
P40763	Q9NQA5	STAT3	TRPV5	26186194;28514442
P40763	Q99062	STAT3	CSF3R	21836163;25402006;9864141
P40763	Q9Y6X2	STAT3	PIAS3	11429412;12804609;18037959;20516148;21836163;21988832;25402006;9388184;9724754;doi:10.1007/978-3-540-73060-6_4
P40763	P50750	STAT3	CDK9	15286705
P40763	Q7L591	STAT3	DOK3	25814554
P40763	Q09472	STAT3	EP300	10205054;11923478;15649887;15653507;15735682;18782771;21836163;22116549;25402006;26504087;doi:10.1007/978-3-540-73060-6_4
P40763	P49137	STAT3	MAPKAPK2	25814554
P40763	Q96DZ7	STAT3	TM4SF19	25814554
P40763	Q6IA86	STAT3	ELP2	10954736;25402006
P40763	P51813	STAT3	BMX	10688651;21516116;25402006;25416956;25814554
P40763	Q3KNS6	STAT3	ZNF829	25814554
P40763	P52333	STAT3	JAK3	10037026;12207328;25402006;doi:10.1007/978-3-540-73060-6_4
P40763	P52294	STAT3	KPNA1	16298512;19084525;21836163;25402006
P40763	Q9Y608	STAT3	LRRFIP2	25609649
P40763	P47712	STAT3	PLA2G4A	23023127;25609649
P40763	Q9NPJ8	STAT3	NXT2	25814554
P40763	Q92905	STAT3	COPS5	21689417;23911788;25402006
P40763	Q96JC1	STAT3	VPS39	25814554
P40763	P51617	STAT3	IRAK1	15465816;25402006
P40763	Q8WTS6	STAT3	SETD7	21098664;25402006
P40763	Q9UMR2	STAT3	DDX19B	26186194;28514442

P40763	Q13547	STAT3	HDAC1	15653507;15870198;22116549;22750444;25402006
P40763	Q8NEC7	STAT3	GSTCD	25814554
P40763	Q8WVB6	STAT3	CHTF18	25814554
P40763	Q9Y3P9	STAT3	RABGAP1	25814554
P40763	Q07666	STAT3	KHDRBS1	11585385;25402006
P40763	Q15642	STAT3	TRIP10	15163742
P40763	P41218	STAT3	MNDA	25814554
P40763	P63000	STAT3	RAC1	11021801;25402006;doi:10.1007/978-3-540-73060-6_4
P40763	Q5TA89	STAT3	HES5	15156153;21836163
P40763	Q15672	STAT3	TWIST1	25814554
P40763	P68402	STAT3	PAFAH1B2	25814554
P40763	Q14847	STAT3	LASP1	25814554
P40763	Q8TE76	STAT3	MORC4	21988832
P40763	Q9P0J0	STAT3	NDUFA13	12628925;12867595;25402006
P40763	Q15788	STAT3	NCOA1	11773079;17471507;25402006
P40763	P23458	STAT3	JAK1	10918587;11294897;11350938;11722592;11751884;11940572;12244095;12576423;12626508;14551213;15284024;25065853;25402006;8272872;
P40763	P24385	STAT3	CCND1	11279133;15659654;20876300;21836163;25402006;doi:10.1007/978-3-
P40763	P19838	STAT3	NFKB1	12057007;25241761;25402006
P40763	P08069	STAT3	IGF1R	11463827;16382134;25241761;25402006
P40763	P07949	STAT3	RET	11536047;12637586;15485908;25241761;25402006
P40763	P18031	STAT3	PTPN1	11970898;15821101;25402006
P40763	P05412	STAT3	JUN	10490649;25402006;doi:10.1007/978-3-540-73060-6_4
P40763	P06401	STAT3	PGR	20876300;21184768;25402006
P40763	P07948	STAT3	LYN	12244095;18070987;23023127;25402006;doi:10.1007/978-3-540-73060-
P40763	O60496	STAT3	DOK2	25402006;25814554
P40763	P01106	STAT3	MYC	20686606;23750211;25402006
P40763	P30084	STAT3	ECHS1	23416296
P40763	P15172	STAT3	MYOD1	12947115;25402006
P40763	P15927	STAT3	RPA2	10875894;25402006;25416956
P40763	O43318	STAT3	MAP3K7	15764709;25402006;3031469
P40763	P26358	STAT3	DNMT1	15870198;25402006;25854163
P40763	O43283	STAT3	MAP3K13	21988832;25402006
P40763	O15379	STAT3	HDAC3	15653507;18037959;22116549;25402006;25892518

P40763	O00459	STAT3	PIK3R2	25402006;25814554
P40763	P27986	STAT3	PIK3R1	23023127;25402006;25814554
P40763	O95661	STAT3	DIRAS3	21643014;21836163
P40763	P35219	STAT3	CA8	25814554
P40763	P22607	STAT3	FGFR3	10918587;11294897;11350938;11940572;12244095;12576423;12626508
P40763	P10275	STAT3	AR	11322786;11751884;12804609;25402006
P40763	P08631	STAT3	HCK	10918587;11294897;11350938;11940572;12244095;12576423;12626508<br>;14551213;21836163;25402006;8626374;doi:10.1007/978-3-540-73060-
P40763	P38936	STAT3	CDKN1A	10764767;20686606;21184768;23750211;25241761;25402006
P40763	P16871	STAT3	IL7R	20167604;7719938
P40763	P22455	STAT3	FGFR4	10918587;11294897;11350938;11940572;12244095;12576423;12626508
P40763	O60674	STAT3	JAK2	10918587;10925297;11294897;11350938;11940572;12244095;12576423<br>;12626508;14551213;15156153;15284024;19834108;21836163;2302312
P40763	O43293	STAT3	DAPK3	16219639;21836163;25402006
P40763	P00533	STAT3	EGFR	10358079;12873986;14963038;14966128;15284024;15485908;15657067<br>;15950906;16273093;18258752;20145033;21573184;21836163;2395613
P40763	P22681	STAT3	CBL	25402006;25814554
P40763	P29590	STAT3	PML	12506013;25402006
P40763	O14874	STAT3	BCKDK	21988832
P40763	P25791	STAT3	LMO2	25416956
P40763	P06239	STAT3	LCK	10825200;18070987;25402006;25814554
P40763	P16591	STAT3	FER	10878010;12738762;25402006
P40763	P17181	STAT3	IFNAR1	16082366;23023127;25402006;8626489;doi:10.1007/978-3-540-73060-
P40763	P27695	STAT3	APEX1	15735682;25402006
P40763	P07384	STAT3	CAPN1	21988832
P40763	O15069	STAT3	NACAD	25814554
P40763	P08047	STAT3	SP1	16931573;17471507;21184768;24396070;25402006
P40763	O60341	STAT3	KDM1A	21098664;25402006
P40763	P04150	STAT3	NR3C1	14522952;25202013;25402006;26508788;9388192;doi:10.1007/978-3-
P40763	P12931	STAT3	SRC	10918587;11294897;11350938;11940572;12244095;12576423;12626508<br>;14551213;15313931;15735682;18070987;19372587;21573184;2183616
P40763	O14512	STAT3	SOCS7	15677474;25402006
P40763	P28482	STAT3	MAPK1	10446219;10521505;11350938;12576423;12763138;14551213;15979846<br>;21836163;25402006;9343414;9872331;doi:10.1007/978-3-540-73060-

P40763	O75695	STAT3	RP2	26186194;28514442
P40763	P27361	STAT3	MAPK3	10446219;10521505;11350938;12576423;12763138;14551213;21836163
P40763	P18847	STAT3	ATF3	25096061;25402006;25814554
P40763	P14625	STAT3	HSP90B1	18662321
P40763	P16234	STAT3	PDGFRA	23023127;25241761;9484840
P40763	P09619	STAT3	PDGFRB	23023127;25241761;25402006;9484840
P40763	P30626	STAT3	SRI	18330356;21988832
P40763	P06454	STAT3	PTMA	15242774;25402006
P40763	P04626	STAT3	ERBB2	11940572;16273093;19372587;20876300;25402006
P40763	O14503	STAT3	BHLHE40	15223310
P40763	P22736	STAT3	NR4A1	21988832;25402006
P40763	P09769	STAT3	FGR	12244095;21988832;25402006;doi:10.1007/978-3-540-73060-6_4
P40763	P20160	STAT3	AZU1	26186194;28514442
P40763	P06400	STAT3	RB1	15677471;25402006
P40763	P31146	STAT3	CORO1A	21988832
P40763	P16949	STAT3	STMN1	16401721;21836163;23333463
Q14765	Q99665	STAT4	IL12RB2	10415122;25402006;9890938
Q14765	Q14765	STAT4	STAT4	14704793;18591661
Q14765	P07550	STAT4	ADRB2	24561123
Q14765	O14796	STAT4	SH2D1B	25814554
Q14765	P46108	STAT4	CRK	25402006;25814554
P42229	Q15303	STAT5A	ERBB4	15534001;16273093;16729043;21836163;22584572;25402006
P42229	Q8N0Z3	STAT5A	SPICE1	26496610
P42229	Q8TC21	STAT5A	ZNF596	21988832
P42229	P52333	STAT5A	JAK3	10037026;18250158;23023127;25402006;9047382;doi:10.1007/978-3-
P42229	Q99081	STAT5A	TCF12	25416956
P42229	Q99490	STAT5A	AGAP2	20075866;25402006
P42229	Q8TAK6	STAT5A	OLIG1	25814554
P42229	Q09472	STAT5A	EP300	14726487;21988832;25402006;26504087;9773981;9989503
P42229	P51692	STAT5A	STAT5B	10358045;16082366;21836163;23023127;25402006;28514442;9398404;
P42229	P46108	STAT5A	CRK	11097834;21836163;25241761;25402006
P42229	P46109	STAT5A	CRKL	11167825;21836163;25402006;9657743;9837784;9872990
P42229	P43365	STAT5A	MAGEA12	16189514
P42229	Q9UEY8	STAT5A	ADD3	26496610

P42229	Q9H0C8	STAT5A	ILKAP	26496610
P42229	Q06124	STAT5A	PTPN11	10617656;12060651;12237455;25402006
P42229	Q99988	STAT5A	GDF15	26496610
P42229	Q9NRG1	STAT5A	PRTFDC1	16189514
P42229	Q99873	STAT5A	PRMT1	18413343
P42229	Q06187	STAT5A	BTK	11413148;23023127;25402006;7925280;8617237;doi:10.1007/978-3-540-
P42229	Q9UMF0	STAT5A	ICAM5	26496610
P42229	Q13287	STAT5A	NMI	16082366;25402006;9989503;doi:10.1007/978-3-540-73060-6_4
P42229	Q9Y2K5	STAT5A	R3HDM2	26496610
P42229	Q9UJU2	STAT5A	LEF1	24394665;25402006
P42229	Q86X55	STAT5A	CARM1	18413343
P42229	Q969D9	STAT5A	TSLP	10570284;11418668
P42229	Q9UGK3	STAT5A	STAP2	15611091;25402006
P42229	P42229	STAT5A	STAT5A	14704793;21988832;9528750
P42229	Q7Z2K6	STAT5A	ERMP1	26496610
P42229	P45973	STAT5A	CBX5	23733954
P42229	O60674	STAT5A	JAK2	11413148;21836163;23023127;25402006;7925280;8617237;9047382;95
P42229	P28482	STAT5A	MAPK1	10194762;10996427;21836163;25241761;25402006
P42229	P10912	STAT5A	GHR	21836163;23023127;25241761;25402006;8702683
P42229	O43567	STAT5A	RNF13	26496610
P42229	P08559	STAT5A	PDHA1	26496610;28514442
P42229	P22681	STAT5A	CBL	12193575;23533197;25402006;25814554
P42229	P09619	STAT5A	PDGFRB	23023127;25241761;25402006;9484840
P42229	P29353	STAT5A	SHC1	10996427;21836163;23397142;25402006
P42229	P03372	STAT5A	ESR1	11682624;15304355;21836163;25402006
P42229	P16410	STAT5A	CTLA4	16476059;23023127;25241761
P42229	P00533	STAT5A	EGFR	10358079;16273093;16729043;22729867;24135280;24658140;25402006
P42229	P10515	STAT5A	DLAT	26496610
P42229	P00367	STAT5A	GLUD1	21988832
P42229	P11177	STAT5A	PDHB	26496610;28514442
P42229	P38159	STAT5A	RBMX	21988832
P42229	O00330	STAT5A	PDHX	26496610
P42229	P18031	STAT5A	PTPN1	12237455;25402006;doi:10.1007/978-3-540-73060-6_4
P42229	O95363	STAT5A	FARS2	26496610

P42229	P01589	STAT5A	IL2RA	11485747
P42229	P16871	STAT5A	IL7R	20167604;23023127;25402006;7719938
P42229	P19235	STAT5A	EPOR	10374881;15644415;21836163;25402006;8977232;doi:10.1007/978-3-
P42229	P23458	STAT5A	JAK1	11722592;25402006;9047382;doi:10.1007/978-3-540-73060-6_4
P42229	P14859	STAT5A	POU2F1	14645506;25402006
P42229	P37231	STAT5A	PPARG	18927468;23223023
P51692	P51692	STAT5B	STAT5B	12089361;14704793;20962278;22729867;9428692
P51692	Q09472	STAT5B	EP300	14726487;25402006;9989503;doi:10.1007/978-3-540-73060-6_4
P51692	Q9UGK3	STAT5B	STAP2	15611091;25402006
P51692	Q06124	STAT5B	PTPN11	10617656;25402006
P51692	Q9P2N6	STAT5B	KANSL3	26496610
P51692	Q9NVP2	STAT5B	ASF1B	26496610
P51692	Q6IA86	STAT5B	ELP2	10954736;25402006
P51692	Q13287	STAT5B	NMI	16082366;25402006;9989503;doi:10.1007/978-3-540-73060-6_4
P51692	Q9H0K6	STAT5B	PUS7L	26496610
P51692	Q14764	STAT5B	MVP	26496610
P51692	P09622	STAT5B	DLD	26496610
P51692	P51681	STAT5B	CCR5	11350939;21836163;25402006
P51692	P19320	STAT5B	VCAM1	19738201;22623428;23023127
P51692	P46108	STAT5B	CRK	10720694;11097834;21836163;25402006;9845531;doi:10.1007/978-3-
P51692	P19235	STAT5B	EPOR	15644415;21836163;25402006;8977232;doi:10.1007/978-3-540-73060-
P51692	P10721	STAT5B	KIT	10358045;17554063;25402006
P51692	P08559	STAT5B	PDHA1	26496610
P51692	P16410	STAT5B	CTLA4	16476059;25241761
P51692	P10912	STAT5B	GHR	21836163;25241761;25402006;8923468
P51692	P14859	STAT5B	POU2F1	14645506;16025120;25402006
P51692	P04150	STAT5B	NR3C1	11158330;21836163;25402006;8878484;9528750;doi:10.1007/978-3-540-
P51692	P06213	STAT5B	INSR	10830280;12456798;21836163;25402006;9122188;9428692
P51692	P18031	STAT5B	PTPN1	12237455;25402006;doi:10.1007/978-3-540-73060-6_4
P51692	P11177	STAT5B	PDHB	26496610
P51692	O00330	STAT5B	PDHX	26496610
P51692	P23458	STAT5B	JAK1	25402006;9047382;doi:10.1007/978-3-540-73060-6_4
P51692	P00533	STAT5B	EGFR	10558875;11751923;12577067;16273093;16729043;22729867;25402006
P51692	P46109	STAT5B	CRKL	10720694;11097834;21836163;25402006;9845531;9872990;doi:10.1007/

P51692	P15822	STAT5B	HIVEP1	26496610
P51692	P40933	STAT5B	IL15	21988832
P51692	O60674	STAT5B	JAK2	12538627;21836163;24354892;25402006;9047382;9575217;doi:10.1007/
P51692	P01589	STAT5B	IL2RA	10602027;11485747;25402006
P51692	P10515	STAT5B	DLAT	26496610
P42226	Q460N5	STAT6	PARP14	16537510
P42226	Q92793	STAT6	CREBBP	10454341;11574547;15695802;25402006
P42226	Q09472	STAT6	EP300	10454341;17237818;23461825;25402006;doi:10.1007/978-3-540-73060-
P42226	P42226	STAT6	STAT6	14704793;22000020;8085155
P42226	Q15788	STAT6	NCOA1	11574547;12138096;14757047;14993689;15695802;18267973;21836163;25402006;doi:10.1007/978-3-540-73060-6_4
P42226	P68104	STAT6	EEF1A1	21988832;26344197
P42226	Q7KZF4	STAT6	SND1	12234934;20225206;25402006
P42226	Q9UHD2	STAT6	TBK1	22000020
P42226	Q15306	STAT6	IRF4	10601358;11342629;25402006
P42226	Q99732	STAT6	LITAF	15793005;25402006
P42226	Q9UBT6	STAT6	POLK	26496610
P42226	Q14807	STAT6	KIF22	26496610
P42226	Q86WV6	STAT6	TMEM173	22000020
P42226	Q7Z434	STAT6	MAVS	22000020
P42226	P52333	STAT6	JAK3	12207328;25402006;doi:10.1007/978-3-540-73060-6_4
P42226	P52630	STAT6	STAT2	10490982;21268015;21836163;25402006;doi:10.1007/978-3-540-73060-
P42226	P23246	STAT6	SFPQ	21106524
P42226	P23458	STAT6	JAK1	10856136;25402006;9651359
P42226	P17181	STAT6	IFNAR1	10490982;25402006
P42226	P37231	STAT6	PPARG	21093321;25402006
P42226	P17706	STAT6	PTPN2	17210636;25402006
P42226	P07355	STAT6	ANXA2	20121258;25402006
P42226	P19838	STAT6	NFKB1	25402006;9584180
P42226	P24394	STAT6	IL4R	15661890;21268015;21836163;23023127;25402006;8816495;9392697;9
