# Supplementary material for: JAK‐STAT core cancer pathway: An integrative cancer interactome analysis
Source: J Cell Mol Med. 2022 Mar 1;26(7):2049–62. doi: 10.1111/jcmm.17228 (PMC8980946; doi:10.1111/jcmm.17228)
Supplement: Supplementary file 7 — Supplementary Material [file JCMM-26-2049-s005.pdf]

Supplementary Figure Captions

Supplementary Figure 1:

STAT interactome with member-specific subgrouping. Each node is a protein, and each edge is an interaction between those proteins replicated either in multiple publications or multiple bioassays. The size of the nodes representing STATs is proportional to their degree (the number of interactions), and the color of each node is representative of the protein's biological process as described in the legend. The length of the edges is arbitrary. Proteins which were found to interact with three or more STATs are grouped in the middle and labeled. Those interacting with two STATs are placed between the respective STATs. A list of references on these PPIs is found in **Supplementary Table 1**.

Supplementary Figure 2:

STAT interactome highlighting the three largest families of protein. Each node is a protein, and each edge is an interaction between those proteins replicated either in multiple publications or multiple bioassays. The size of the nodes is proportional to their degree (the number of interactions), and the color of each node is representative of the protein's function assigned based on PANTHER (reference: Mi H, Ebert D, Muruganujan A, Mills C, Albou LP, Mushayamaha T, Thomas PD. PANTHER version 16: a revised family classification, tree-based classification tool, enhancer regions and extensive API. Nucleic Acids Res. 2021 Jan 8;49(D1):D394-D403. doi: 10.1093/nar/gkaa1106. PMID: 33290554; PMCID: PMC7778891) protein class and protein family/subfamily as described in the legend. Some proteins with multiple and/or equivocal functions were placed under the "Other" category. The length of the edges is arbitrary. Proteins which were found to interact with three or more STATs are grouped in the middle and labeled. A list of references on these PPIs is found in **Supplementary Table 1**.

Supplementary Figure 3:

STAT Interactome filtered by cell component. STAT interactions have been segregated on localization in the cytoplasm, nucleus, mitochondrion, and plasma membrane in panels into panels A, B, C and D, respectively.

Supplementary Figure 4:

STAT Interactome filtered by blood cancers. Proteins which were found to interact with three or more STATs are grouped in the middle and labeled. Those interacting with two STATs are placed between the respective STATs. Any interaction shared between two STATs or more is grouped in the middle and the edges going to those interaction are highlighted. Proteins for which a label is shown are those which are reported to be over-expressed in that disease.

Supplementary Figure 5:

STAT interactors filtered by gene classification. The network depicts STAT interaction with proteins whose encoding genes are either classified as oncogenes or tumor suppressors. The ratios of the number of oncogenes to tumor suppressors that interact with STAT1, STAT2, STAT3, STAT5A, STAT5B and STAT6 are 2.4, 1, 1.75, 4.5, 5 and 2.5, respectively.
